# Supplementary material for: Cell-type-directed design of synthetic enhancers
Source: Nature. 2023 Dec 12;626(7997):212–20. doi: 10.1038/s41586-023-06936-2 (PMC10830415; doi:10.1038/s41586-023-06936-2)
Supplement: Supplementary file 1 — Supplementary Notes and Figures. Notes: validation of motif level explainability of deep learning models, enhancer design by GANs and assessment of the activator/repressor balance in human enhancers. Figs. 1–16: nucleotide contribution scores of all sequences not already displayed in main text or Extended Data accompanied eventually of in silico saturation mutagenesis prediction scores. Plots of prediction scores for the selection of mutations for the dual-code enhancers. [file 41586_2023_6936_MOESM1_ESM.pdf]

nature portfolio

https://doi.org/10.1038/s41586-023-06936-2

# Supplementary information

# Cell-type-directed design of synthetic enhancers

In the format provided by the authors and unedited

## Supplementary Note 1: Validation of motif level explainability of deep learning models

In this work we have used a combination of DeepExplainer, in silico mutagenesis, perturbation experiments, and experimental validations to validate specific motif-level predictions. Despite the terms model interpretability or explanations, DeepExplainer does not provide a full explanation of model predictions. Indeed, DeepExplainer provides independent nucleotide contributions, not motif level contribution scores. Therefore, when using DeepExplainer for observational analyses of candidate TF binding sites, it is important to validate the enhancer model and to complement DeepExplainer-based attribution scores with independent measurements or analyses.

The two deep neural network models we use in this work are DeepFlyBrain and DeepMEL2. Both models were subject to rigorous validation in previous publications[8,37,39,76] where we assessed whether DeepExplainer-based predictions, which from an observational viewpoint provide predictions of TF motifs' contribution to enhancer activity, are accurate. By combining multiple lines of direct as well as circumstantial evidence, the confidence in motif-level predictions can be increased.

### DeepExplainer-based attribution scores correlate with saturation mutagenesis, ChromBPNet, and Enformer

For nucleotide-level importances and motif-level interpretations of human MEL enhancers we compared three models, each with DeepExplainer and in silico mutagenesis, on genomic enhancers (e.g. IRF4 enhancer) and synthetic enhancers. For the genomic IRF4 enhancer we additionally compare all these nucleotide-level importances with experimental saturation mutagenesis[84] (Supplementary Note 1 – Figure 1).

For both genomic and synthetic enhancers, the DeepExplainer-based importance scores agree with all other independent measurements and predictions. Particularly, the identified ‘seqlets’, by using TF-Modisco (blocks of nucleotides with high DeepExplainer importance scores), that correspond to candidate TF binding sites for SOX10, TFAP2, MITF, and ZEB2 agree between the models, both for genomic and for synthetic enhancers.

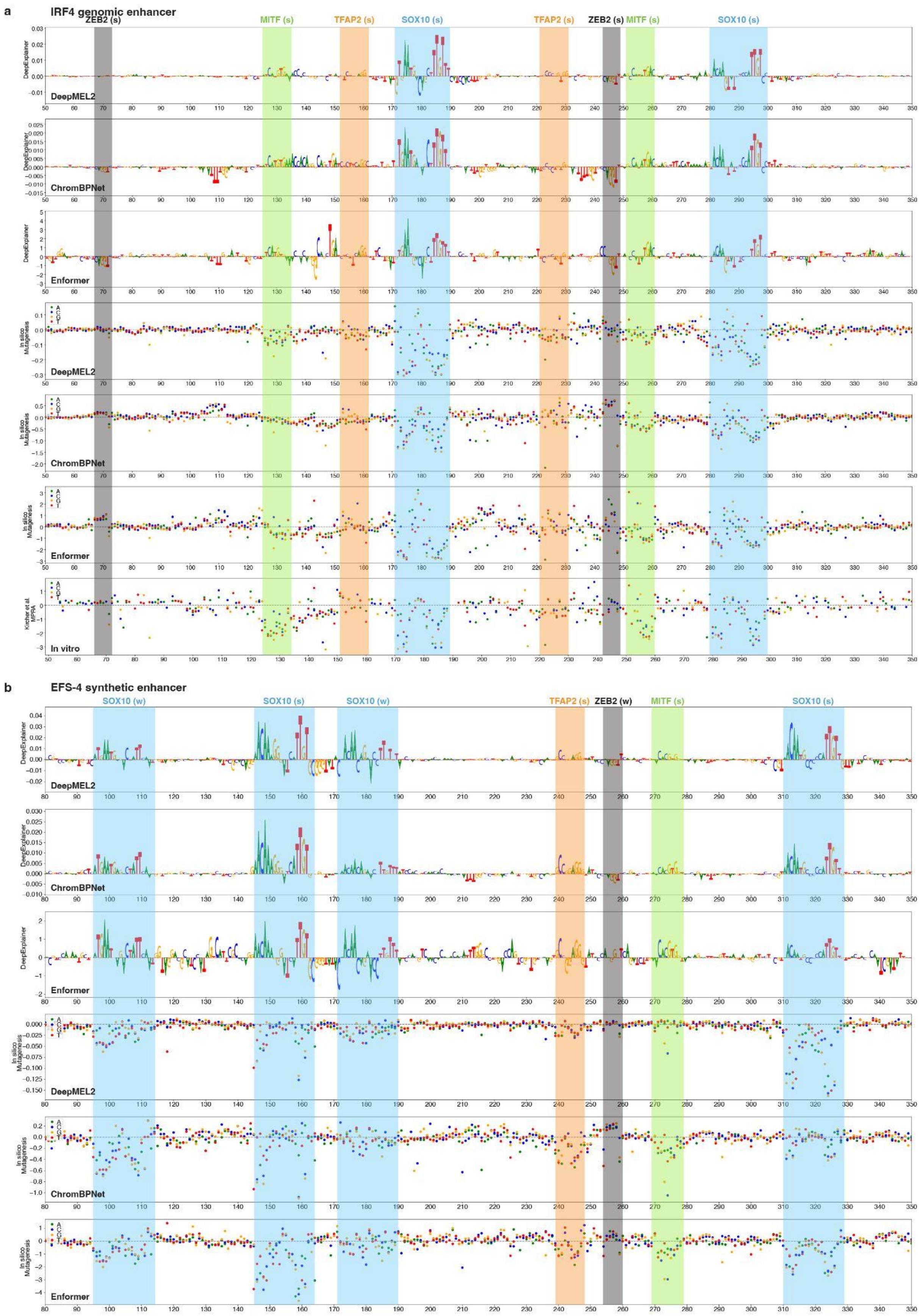


**Supplementary Note 1 – Figure 1: Comparison of DeepExplainer and in silico mutagenesis from different models**

Nucleotide contribution scores and in silico saturation mutagenesis assays of DeepMEL2, ChromBPNet, and Enformer for IRF4 (**a**) and EFS-4 (**b**). In vitro saturation mutagenesis from Kircher et al.[84] is also displayed for IRF4. Each dot on the

saturation mutagenesis plots represents a single mutation and its effect on the prediction score ($y$ axis). Motif annotation is indicated with strong (s) or weak (w) motif instances.

**Cell-type specific co-expressed TFs correspond to enriched patterns for the same cell type.** By collecting all "seqlets" - these are blocks of nucleotides with high attribution scores, and clustering them using TF-Modisco, patterns are discovered that represent recurrent seqlets across a set of chosen ATAC-seq peaks. For example, when TF-Modisco was applied to all seqlets predicted on all MEL enhancers, the recurrent motifs correspond exactly to the recognition sequences of SOX10, MITF, and TFAP2 for activators, and to ZEB1/ZEB2 for the main repressor motif (based on comparison to position weight matrices from motif databases, see Extended Data Fig. 1g and Extended Data Fig. 5c). These activator TFs are uniquely co-expressed in melanocytes and melanocyte-like melanoma. Likewise, the seqlets obtained from all high-scoring regions of three Kenyon cell subtypes, glial cell types, and T-neuron types in the fly brain lead to enriched motifs by TF-Modisco, matching exactly the recognition sequences of the TFs that are combinatorially uniquely expressed in the respective cell types. This is shown in the 'code tables' of Janssens et al.[39].

**Patterns identified from DeepExplainer/TF-Modisco are confirmed by TF binding experiments (Cut&Tag, ChIP-seq, and DamID).** The recurring patterns identified by DeepExplainer/TF-Modisco for DeepMEL2 and DeepFlyBrain are not only corresponding to the expected expressed TF combination, but these patterns (motifs) could also be confirmed by ChIP-seq (SOX10, MITF, AP-1, ZEB2) in the case of DeepMEL2[37]; and by Cut&Tag for Eyeless in Kenyon cells[39] and by DamID for Mef2 in Kenyon cells[39]. Note that due to the noise and sensitivity of these experiments, not every motif *instance* can be validated (this is a general challenge in the field, and the reason why computational models are developed), but the motif (pattern) can.

**Synthetically designed enhancers are enriched for the same patterns, by different approaches.** When enhancers are designed by in silico evolution, starting from thousands of random sequences, the high-scoring sequences (that could be validated in vitro and in vivo as an entire sequence) show instances of the same patterns as found in genomic enhancers. TF-Modisco identifies the same recurrent patterns from the seqlets in synthetic enhancers, as from the seqlets in genomic enhancers[8,39]. Furthermore, when these synthetic enhancers are analyzed by classical motif enrichment tools that are independent of DeepExplainer (e.g., position weight matrix (PWM) scanning using hidden markov models (HMMs)), the same motifs are enriched as in genomic enhancers of the respective cell type (e.g., SOX10, MITF, TFAP2 for designed MEL enhancers, and Ey/Mef2/Onecut for Kenyon cell enhancers) and depleted for the repressor motifs (ZEB for MEL and Mamo for Kenyon cells).

**Motif-embedding yields expected DeepExplainer predictions.** When instances of known motifs are embedded in random sequences, whereby the best location is chosen by the model (without using DeepExplainer), then the DeepExplainer attribution scores correspond to the inserted motif position. Examples of this are shown in Figure 4e, Extended Data Fig. 4g, and Extended Data Fig. 10c.

**Experimental validation of motif-level predictions.** In previous publications, DeepExplainer-based motif predictions on genomic enhancers (derived by the DeepMEL2 or DeepFlyBrain models) were validated using various motif-level validations[8,39,76]. For example, mutations in predicted Mef2 and Ey

sites abolished in vivo enhancer activity; mutations in predicted Mamo/lola repressor sites increased enhancer activity; mutations in predicted SOX10 and MITF sites reduce in vitro enhancer activity; embedding of SOX10 and MITF sites in random sequences create active enhancers; adding ZEB sites to genomic or synthetic MEL enhancers abolishes enhancer activity.

**Supplementary Note 2: Enhancer design by Generative Adversarial Networks**

We trained a GAN on genomic sequences, using KC enhancers as a training set. After training, we used the generator part of the model to generate synthetic sequences (Methods). To evaluate the GAN-generated sequences we used the DeepFlyBrain model to compare the generated sequences with randomly generated sequences using a Markov model (Methods). After 20k batch iterations, the GAN model started outperforming a 5th-order background model (Supplementary Note 2 - Fig. 1a). Moreover, the GAN model captured high-order genomic sequence features (e.g. GC content is higher in the centre) while the background model failed as it only focuses on k-mer frequencies and fails to capture any location bias (Supplementary Note 2 - Fig. 1b). However, sequence prediction scores did not reach a level as high as genomic KC enhancers (Supplementary Note 2 - Fig. 1c). Calculating nucleotide contribution scores of the generated sequences revealed relevant binding sites which appear in a similar frequency to genomic regions, although these sites were weaker compared to the sequences generated by the first two strategies (Supplementary Note 2 - Fig. 1d,e). We selected 5 of the generated sequences that were predicted as KC enhancers and harboured a combination of weak/strong Ey, Mef2, Onecut and Sr binding sites (Supplementary Fig. 16). Testing their activity in vivo showed that the sequences with better binding sites drove clearer GFP expression in KC (Supplementary Note 2 - Fig. 1f). In conclusion, GAN-generated sequences can also produce functional and cell type specific enhancers but require a posteriori filtering with pre-trained enhancer models.

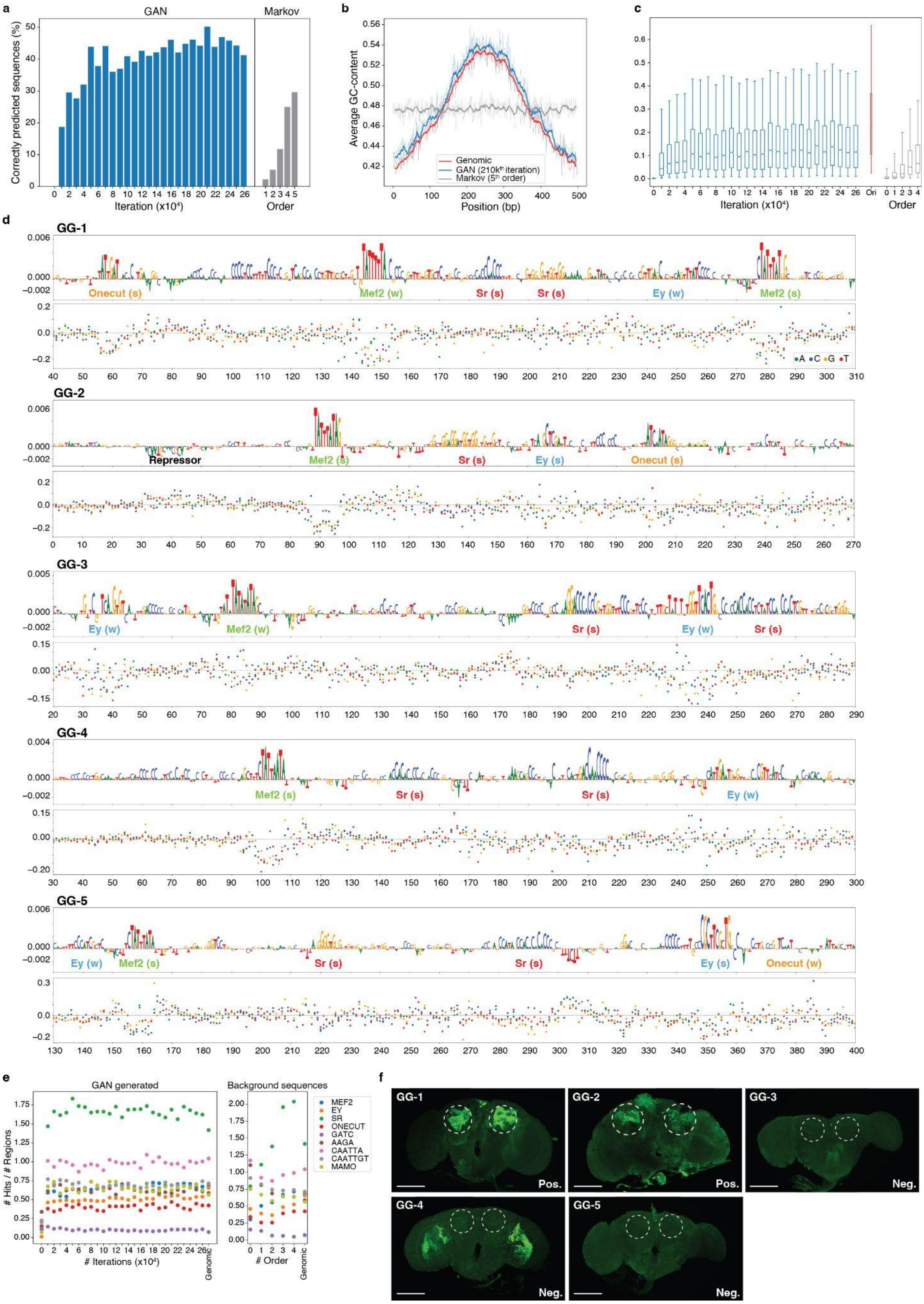


**Supplementary Note 2 - Figure 1: Generative design of fly enhancers**

**a**, Percentage of correctly predicted sequences (prediction score > 0.25) that are generated after each 10,000 batch iterations of the GAN model ($n$ = 6144 for each iteration) and that are generated by a Markov model with different orders ($n$ = 6144 for each order). **b**, Average GC-content over 500 bp genomic ($n$ = 6126), GAN-generated at 210k$^{th}$ batch iteration ($n$

= 6144), and 5$^{th}$ order Markov-generated sequences ($n$ = 6144). **c**, Prediction score distribution of the generated sequences sampled at different iterations of the GAN training (in blue, $n$ = 6144 sequences), original genomic sequences that are used to train the GAN models (red, $n$ = 6126 sequences), and background sequences that are generated with different order of k-mer compositions (grey, $n$ = 6144 sequences) for KC class. The box plots show the median (center line), interquartile range (box limits), and 5th and 95th percentile range (whiskers). **d**, Nucleotide contribution scores of selected GAN-generated sequences (top) and their in silico saturation mutagenesis assays (bottom). Each dot on the saturation mutagenesis plot represents a single mutation and its effect on the prediction score ($y$ axis). Motif annotation is indicated with strong (s) or weak (w) motif instances. **e**, Average number of motif hits at each GAN iteration compared to Genomic enhancers and Markov model. **f**, In vivo enhancer activity of the cloned GAN-generated sequences. The expected location of KC is shown with dashed circles. GG: GAN Generated.

We tested the GAN based sequence generation approach in our human model. Very similar to what we observed in the *Drosophila* case, the GAN model started outperforming background models after 20k batch iterations and again captured the high-order sequence features, as well as motif content, much better than a Markov model (Supplementary Note 2 - Fig. 2a-d). We selected 10 GAN-generated sequences (GG1-10) from the best batch iteration that contain SOX10, MITF, and TFAP2 binding sites (Supplementary Note 2 - Fig. 2e). Testing them in vitro with a luciferase assay showed that five of them were active in the range of genomic enhancers (Supplementary Note 2 - Fig. 2f). Observed activity levels in a mesenchymal-like (MES) melanoma line were overall higher (meaning, their cell type specificity is lower) for the GAN-generate enhancers, compared to the enhancers designed by in silico sequence evolution or motifs implantation. Nevertheless, some of the most active GAN-generated sequences in the MEL line (e.g. GG-3 and GG-4) are highly specific as they have very low activity in this MES line (Supplementary Note 2 - Fig. 2g).

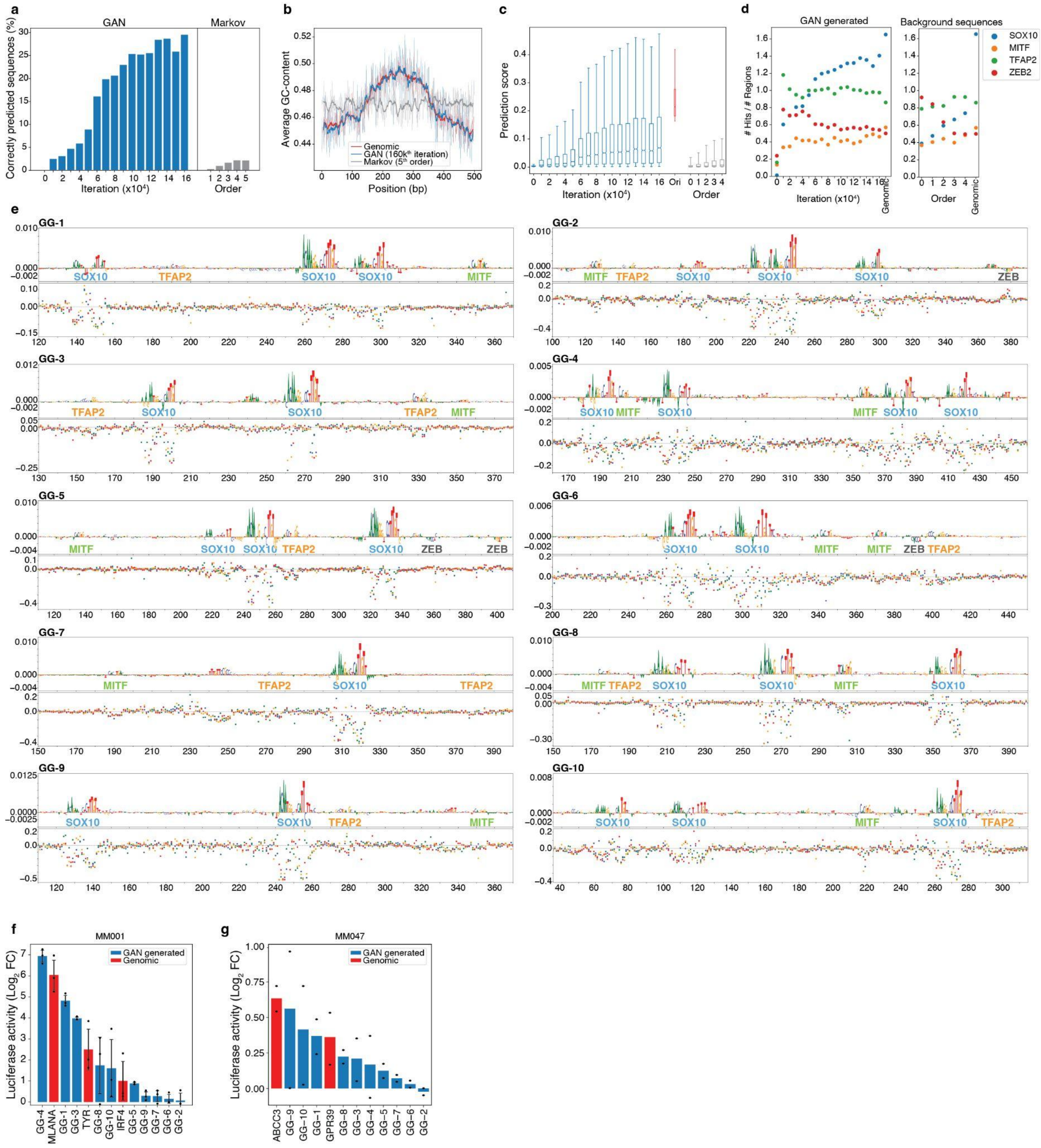


**Supplementary Note 2 - Figure 2: Human enhancer design by Generative Adversarial Networks**

**a**, Percentage of correctly predicted sequences (prediction score > 0.15) that are generated after each 10,000 batch iteration of the GAN model ($n$ = 3968 for each iteration) and that are generated by Markov model with different orders ($n$ = 3968 for each order). **b**, Average GC-content over 500 bp genomic ($n$ = 3885), GAN-generated at 160k$^{th}$ batch iteration ($n$ = 3968), and 5$^{th}$ order Markov-generated sequences ($n$ = 3968). **c**, Prediction score distribution of the generated sequences sampled at different iterations of the GAN training (in blue, $n$ = 3968), original genomic sequences that are used to train the GAN models (red, $n$ = 3885), and background sequences that are generated with different order of k-mer compositions (grey, $n$ = 3968) for MEL class. The box plots show the median (center line), interquartile range (box limits), and 5th and 95th percentile range (whiskers). **d**, Average number of motif hits at each GAN iteration compared to Genomic enhancers and Markov model. **e**, Nucleotide contribution scores of the selected GAN-generated sequences (top) and their in silico saturation mutagenesis assays (bottom). Each dot on the saturation mutagenesis plots represents a single mutation and its effect on the prediction score ($y$ axis). The name of the identified binding sites is written in between top and bottom plots. **f**, Bar plot showing the mean luciferase signal ($\log_2$ fold-change over *Renilla*) of the GAN-generated sequences and genomic enhancers and genomic MEL enhancers in MM001 (MEL melanoma line). The error bars show the standard error of the mean ($n$ = 3 biological replicates). **g**, Bar plot showing the mean luciferase signal ($\log_2$ fold-change over *Renilla*) of the

GAN-generated sequences and genomic MES enhancers in MM047 (MES melanoma line). The bars show the mean ($n$ = 2 biological replicates).

GANs have proven their strengths in the generation of realistic samples in many different fields and they have been proposed to generate enhancer sequences in silico[46]. In our experience, by only using around 4,000-6,000 genomic sequences as training samples, GAN models successfully generated sequences of which around 30-50% were correctly predicted to be specific for the trained class. This lower success rate is also reflected in lower prediction scores obtained by the generated sequences. This might be further improved in the future by using a larger set of real samples (e.g., cross-species and multiple cell types) or by improving network architectures. Even though GANs could generate functional enhancers, we needed a trained deep learning model to measure the quality of the generated sequences. To our knowledge, there is no simple technique to measure the performance of the generator and the discriminator during training. In this aspect, sequence evolution and motif implantation approaches were more interpretable and straightforward compared to generative design.

## Supplementary Note 3: Assessment of the activator/repressor balance in human enhancers

In the light of the results on *Drosophila* enhancers, we investigated whether the interpretation of human enhancers, using deep learning models, follows the same principles as *Drosophila* enhancers, particularly regarding the balance between activator and repressor motifs. To this end, we used our previously trained and validated melanoma deep learning model, DeepMEL2[8,76] (Supplementary Note 1), to study an intronic enhancer of the IRF4 gene, for which in vitro saturation mutagenesis data is available[84] (Supplementary Note 3 - Fig. 1a). For this melanocyte, or melanocyte-like melanoma (MEL) enhancer, candidate TF binding sites are revealed by DeepExplainer attribution scores for SOX10, MITF, and TFAP2A with positive contribution (suggestive of activator roles), and for a ZEB family member (e.g., ZEB1 or ZEB2) with negative contribution (suggestive of a repressor role). In silico saturation mutagenesis on this enhancer correlates significantly (Spearman = 0.646, Fig. 5i) with massively parallel reporter assays data (Supplementary Note 3 - Fig. 1a). The delta prediction scores suggest that the activity of this enhancer decreases by mutating the activator sites. However, many isolated mutations are also predicted to decrease the activity and result in the creation of ZEB repressor sites (Supplementary Note 3 - Fig. 1b). We then employed two independent deep learning models, namely a newly trained ChromBPNet model[50] on bulk MM001 ATAC-seq data and the previously published Enformer model, for which the SK-MEL-5 ATAC-seq class represents the MEL state[6]. Both these models agree with DeepMEL2 explanations in terms of activator and repressor sites and show similar predictions of perturbations: mutations that generate ZEB repressor sites lead to decreased activity (similarly to mutating the activator sites of SOX10 or MITF); while mutations that destroy the endogenous ZEB site lead to increased activity (Supplementary Note 3 - Fig. 1c,e, Supplementary Note 3 - Fig. 2a). To experimentally validate these findings, we mutated the ZEB sites in the IRF4 enhancer, and observed a 4-fold increase in enhancer activity using luciferase assays (Supplementary Note 3 - Fig. 1c, Supplementary Note 3 - Fig. 2b). Conversely, when we mutated the enhancer to generate additional ZEB sites, the enhancer activity was completely abolished, similarly to the mutation of the SOX10 or MITF sites (Supplementary Note 3 - Fig. 1c, Supplementary Note 3 - Fig. 2b).

This result suggests that a repressor TF is acting on this enhancer in the MEL cells themselves. To verify this, we used previously published scRNA-seq data on a cohort of melanoma cell lines, and found that ZEB2 is expressed in the MM001 MEL line, while ZEB1 is not expressed in MEL lines[82]. We performed ChIP-seq against ZEB2 and found a significant ChIP-seq peak on the IRF4 enhancer, coinciding with a SOX10 ChIP-seq peak, suggesting that both activators and repressors can bind to this enhancer (Fig. 5g). This enhancer is only moderately active as shown by the luciferase reporter above, but still accessible. Regions with very high ZEB2 ChIP-seq peaks (and more ZEB motif instances compared to the IRF4 enhancer), on the other hand, are not accessible (Fig. 5g, Supplementary Note 3 - Fig. 1d, Supplementary Note 3 - Fig. 2c). This further supports our model that ZEB repressor sites, like the *Drosophila* repressors identified above, correlate with lower accessibility, and activity. Furthermore, the Enformer model allowed us to assess the impact of these enhancer mutations on the expression of the IRF4 target gene (using SK-MEL-5 CAGE data) and on the changes of chromatin accessibility and H3K27Ac (Fig. 5h), which are all congruent with the predicted changes in enhancer activity. These findings, for this case study of a melanocyte-like enhancer, suggest that human and *drosophila* enhancer logic follow similar principles and that our deep learning models capture their essential architecture. We, thus, proceeded with human enhancer design along the same principles as we did above for the fly brain.

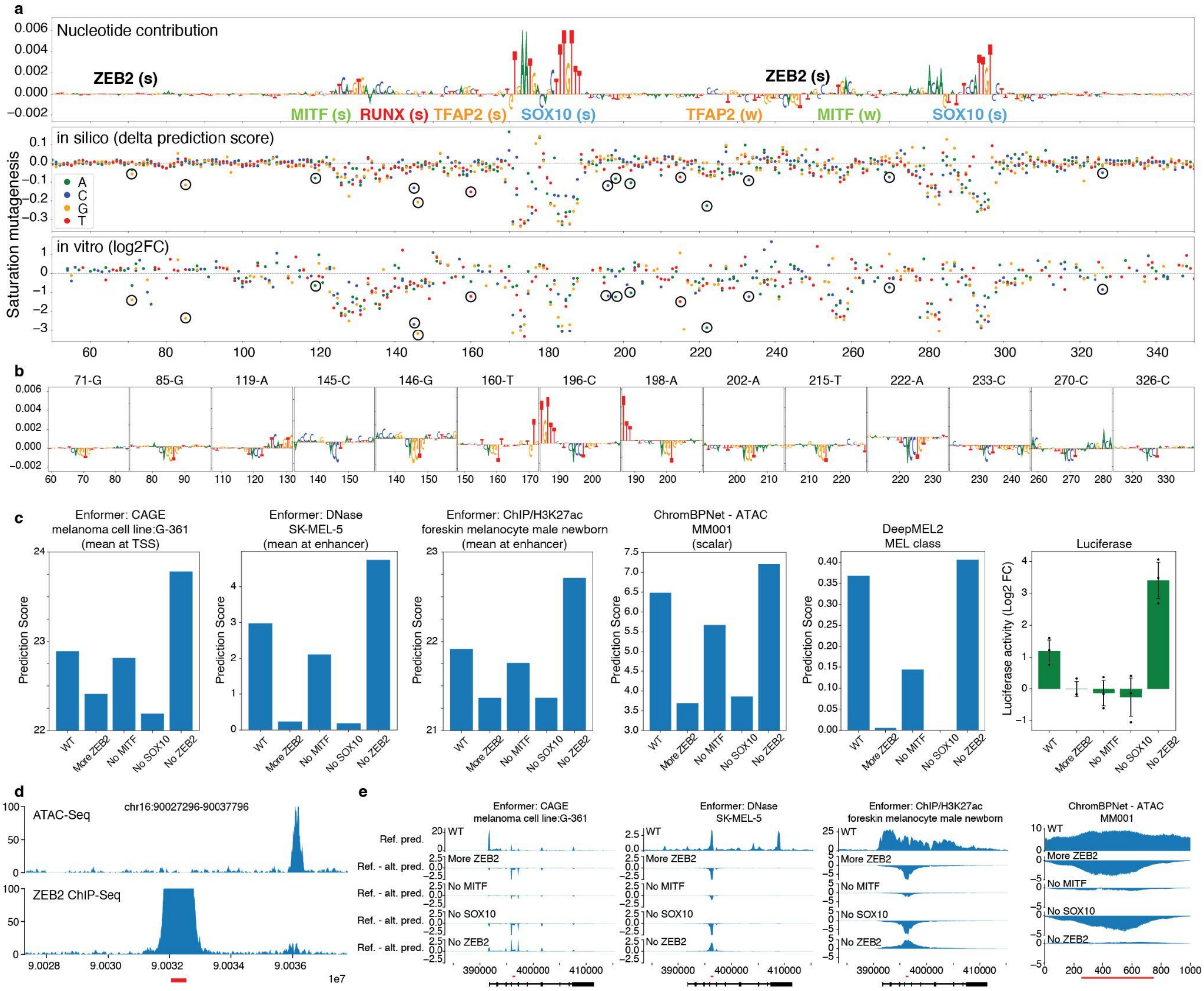


**Supplementary Note 3 - Figure 1: Human melanocyte-like enhancer contains both functional activator and repressor motifs**

**a**, Nucleotide contribution score (top) and in silico saturation mutagenesis (middle) of the IRF4 enhancer for DeepMEL2 human melanocyte-like melanoma class. In vitro saturation mutagenesis in SK-MEL-28 (bottom). Mutations resulting in a drop of both prediction and activity, and that generate a ZEB2 binding site are highlighted with a black circle. **b**, Nucleotide contribution scores of newly generated ZEB2 motifs from mutations highlighted in panel **a**. **c**, First five panels: prediction scores for three different Enformer classes, ChromBPNet trained on MM001 ATAC-seq, and DeepMEL2 MEL class for the WT IRF4 enhancer and mutant versions. Right panel: luciferase activity of the WT IRF4 enhancer and mutant versions in MM001. Error bars in the right panel denote mean standard error ($n$ = 3 biological replicates). **d**, ATAC-seq (top) and ZEB2 ChIP-seq (bottom) tracks in MM001 highlight the chr16:90027296-90037796 region (red bar). **e**, First three panels: Enformer prediction tracks for three classes for the IRF4 enhancer. Top track: WT IRF4 enhancer prediction score, other tracks: delta of mutated IRF4 enhancer prediction score vs WT IRF4 enhancer prediction score. Right panel: ChromBPNet trained on MM001 ATAC-seq prediction tracks for the same sequences. The IRF4 enhancer location is highlighted in red.

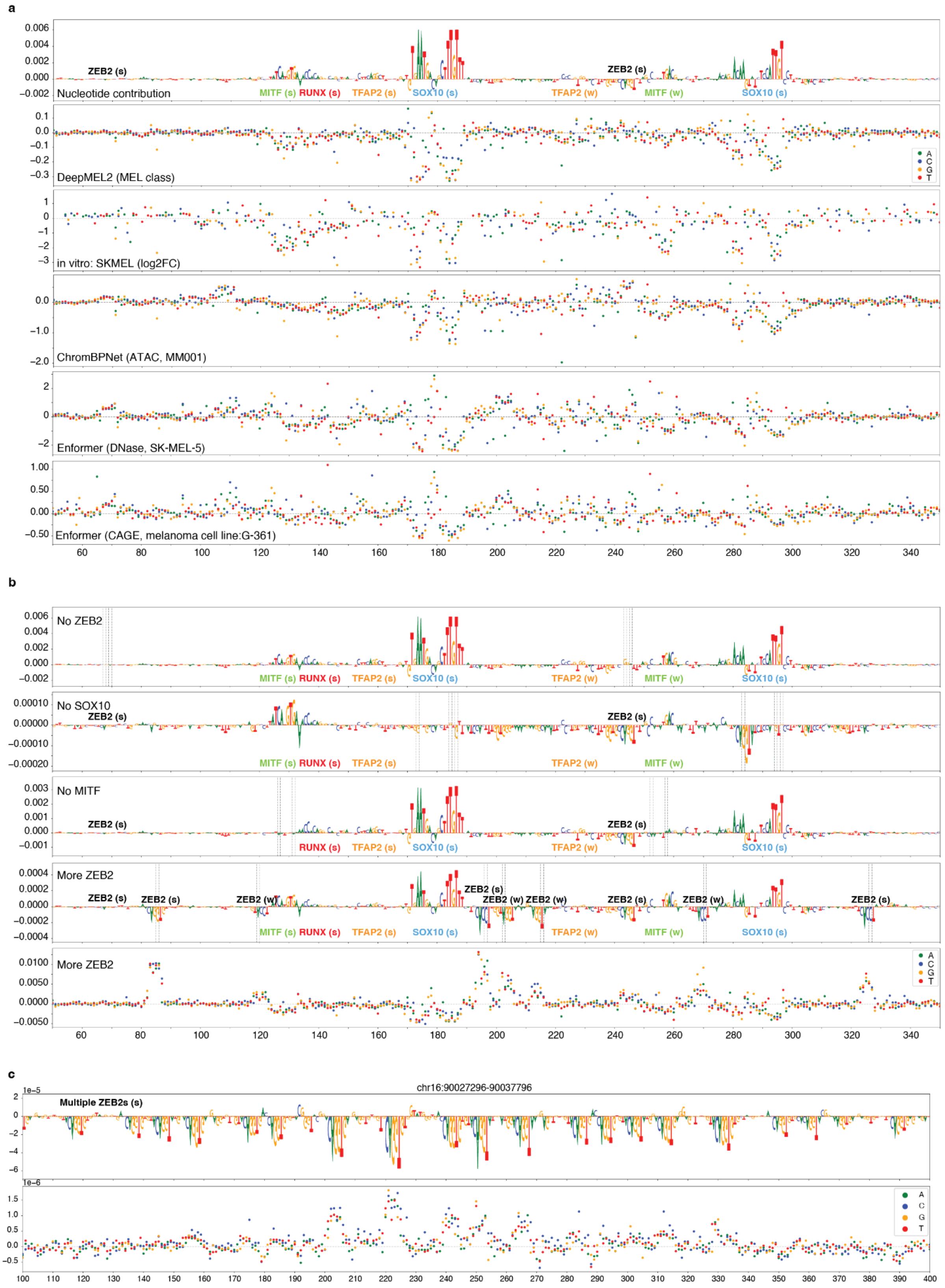


**Supplementary Note 3 - Figure 2: ZEB2 repressor activity in human melanocyte-like melanoma enhancer**

**a**, DeepMEL2 nucleotide contribution score (first track) and in silico and in vitro saturation mutagenesis for the IRF4 enhancer. **b**, DeepMEL2 nucleotide contribution scores (first four tracks) for mutant versions of the wild-type (WT) IRF4 enhancer. In silico saturation mutagenesis of the IRF4 enhancer with additional ZEB2 sites (bottom track). Mutations introduced to the WT enhancer are indicated by dashed lines. **c**, Nucleotide contribution score (top) and in silico saturation

mutagenesis (bottom) of ZEB2 ChIP-seq high region (chr16:90027296-90037796) for the MEL class. In panels **a**, **b**, **c**, motif annotation is indicated with strong (s) or weak (w) motif instances.

## References:


6. Avsec, Ž. *et al.* Effective gene expression prediction from sequence by integrating long-range interactions. *Nat Methods* **18**, 1196–1203 (2021).

8. Atak, Z. K. *et al.* Interpretation of allele-specific chromatin accessibility using cell state-aware deep learning. *Genome Res.* gr.260851.120 (2021) doi:10.1101/gr.260851.120.

37. Minnoye, L. *et al.* Cross-species analysis of enhancer logic using deep learning. *Genome Res.* **30**, 1815–1834 (2020).

39. Janssens, J. *et al.* Decoding gene regulation in the fly brain. *Nature* 1–7 (2022) doi:10.1038/s41586-021-04262-z.

46. Zrimec, J. *et al.* Controlling gene expression with deep generative design of regulatory DNA. *Nat Commun* **13**, 5099 (2022).

50. Brennan, K. J. *et al.* Chromatin accessibility in the Drosophila embryo is determined by transcription factor pioneering and enhancer activation. *Developmental Cell* **58**, 1898-1916.e9 (2023).

76. Mauduit, D. *et al.* Analysis of long and short enhancers in melanoma cell states. *eLife* **10**, e71735 (2021).

64. Shrikumar, A., Greenside, P. & Kundaje, A. Learning Important Features Through Propagating Activation Differences. Preprint at https://doi.org/10.48550/arXiv.1704.02685 (2019).

65. Lundberg, S. M. & Lee, S.-I. A unified approach to interpreting model predictions. in *Proceedings of the 31st International Conference on Neural Information Processing Systems* 4768–4777 (Curran Associates Inc., 2017).

82. Wouters, J. *et al.* Robust gene expression programs underlie recurrent cell states and phenotype switching in melanoma. *Nature Cell Biology* **22**, 986–998 (2020).

84. Kircher, M. *et al.* Saturation mutagenesis of twenty disease-associated regulatory

elements at single base-pair resolution. *Nat Commun* **10**, 3583 (2019).

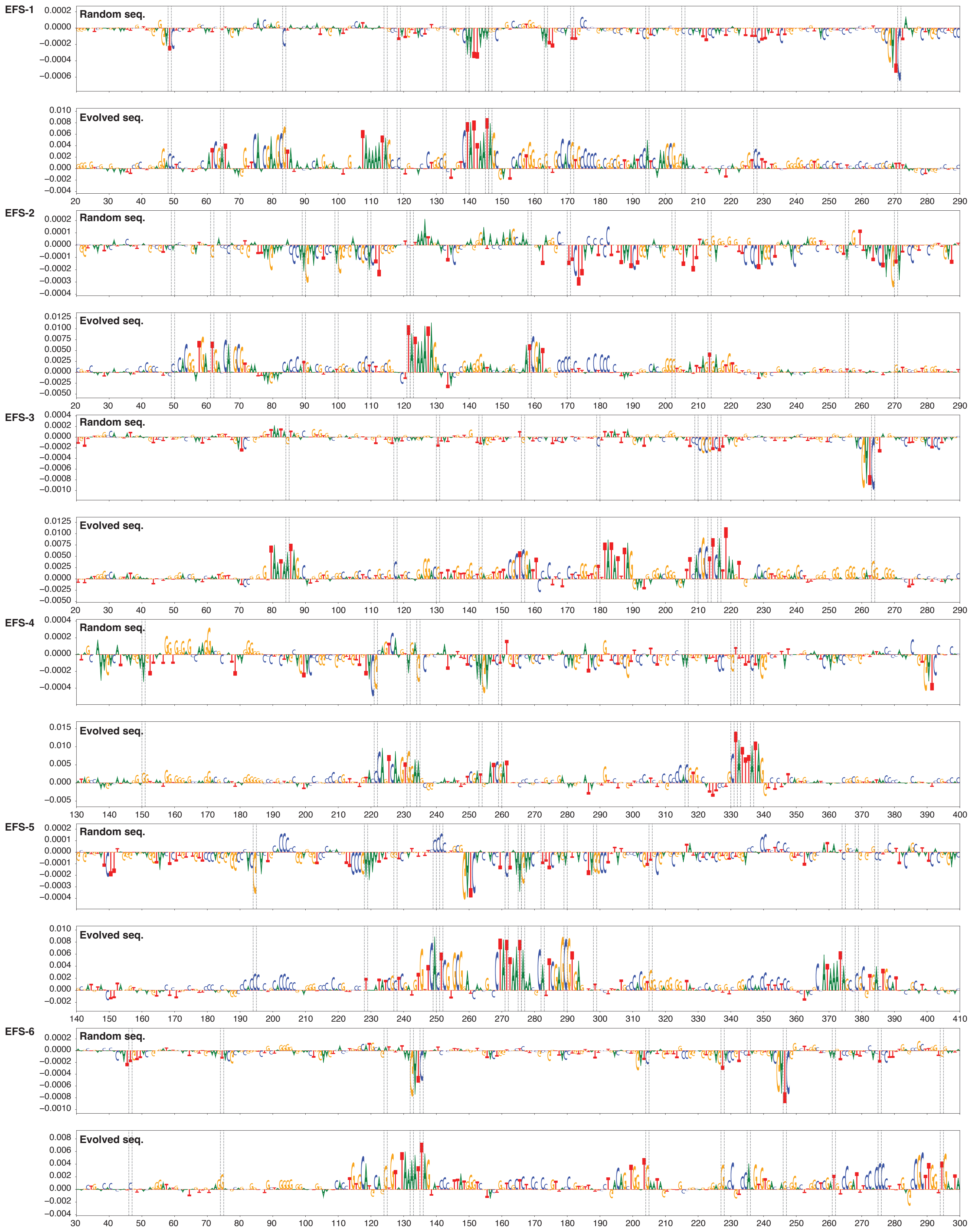


**Supplementary Figure 1: Kenyon cell in silico evolution (EFS-1 to EFS-6)**
Nucleotide contribution score (DeepFlyBrain KC class) of the original random sequence (top) and evolved sequence (bottom) for each selected in silico evolution sequence. Location of mutated nucleotides is indicated by dashed lines.

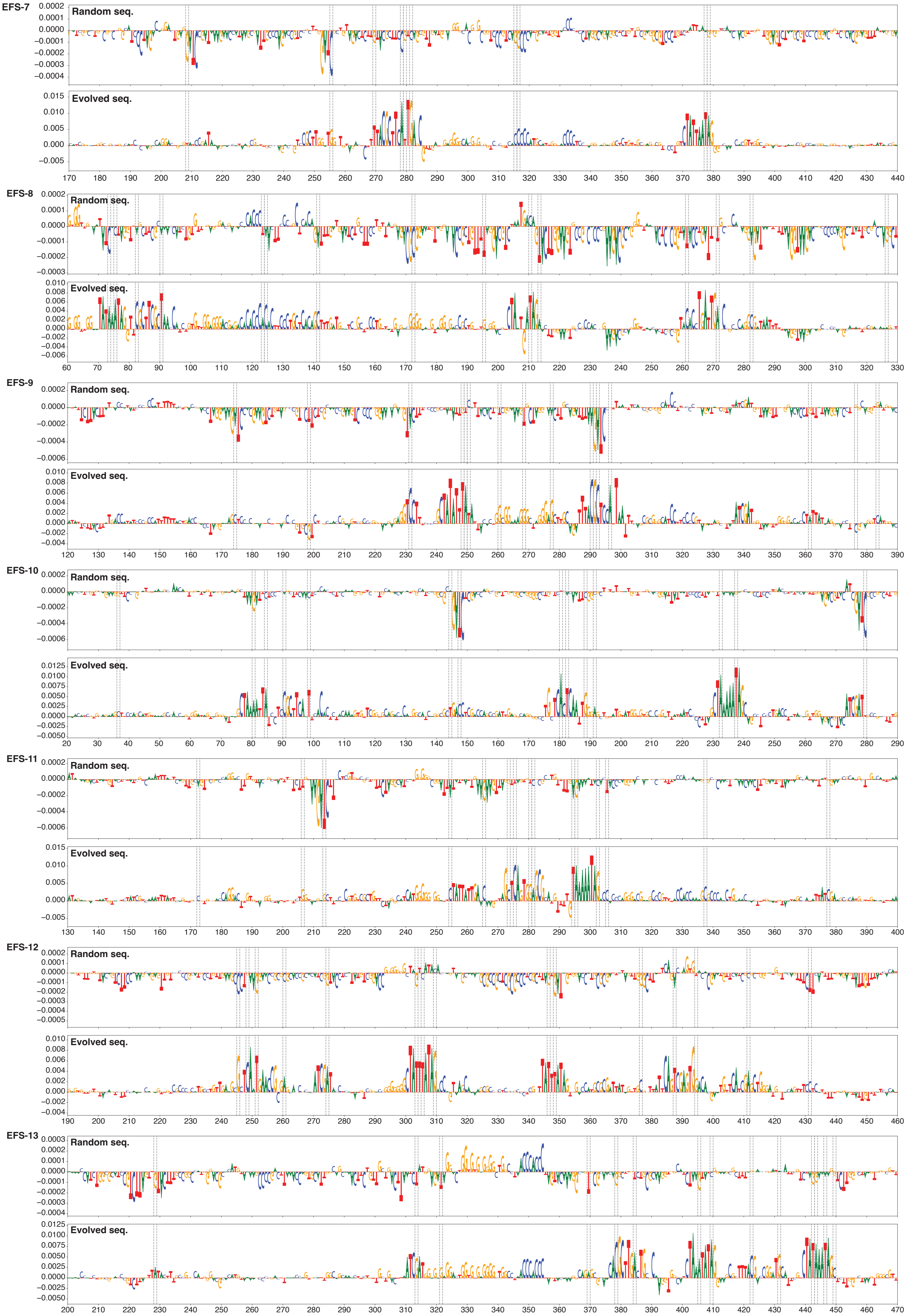


**Supplementary Figure 2: Kenyon cell in silico evolution (EFS-7 to EFS-13)**

Nucleotide contribution score (DeepFlyBrain KC class) of the original random sequence (top) and evolved sequence (bottom) for each selected in silico evolution sequence. Location of mutated nucleotides is indicated by dashed lines.

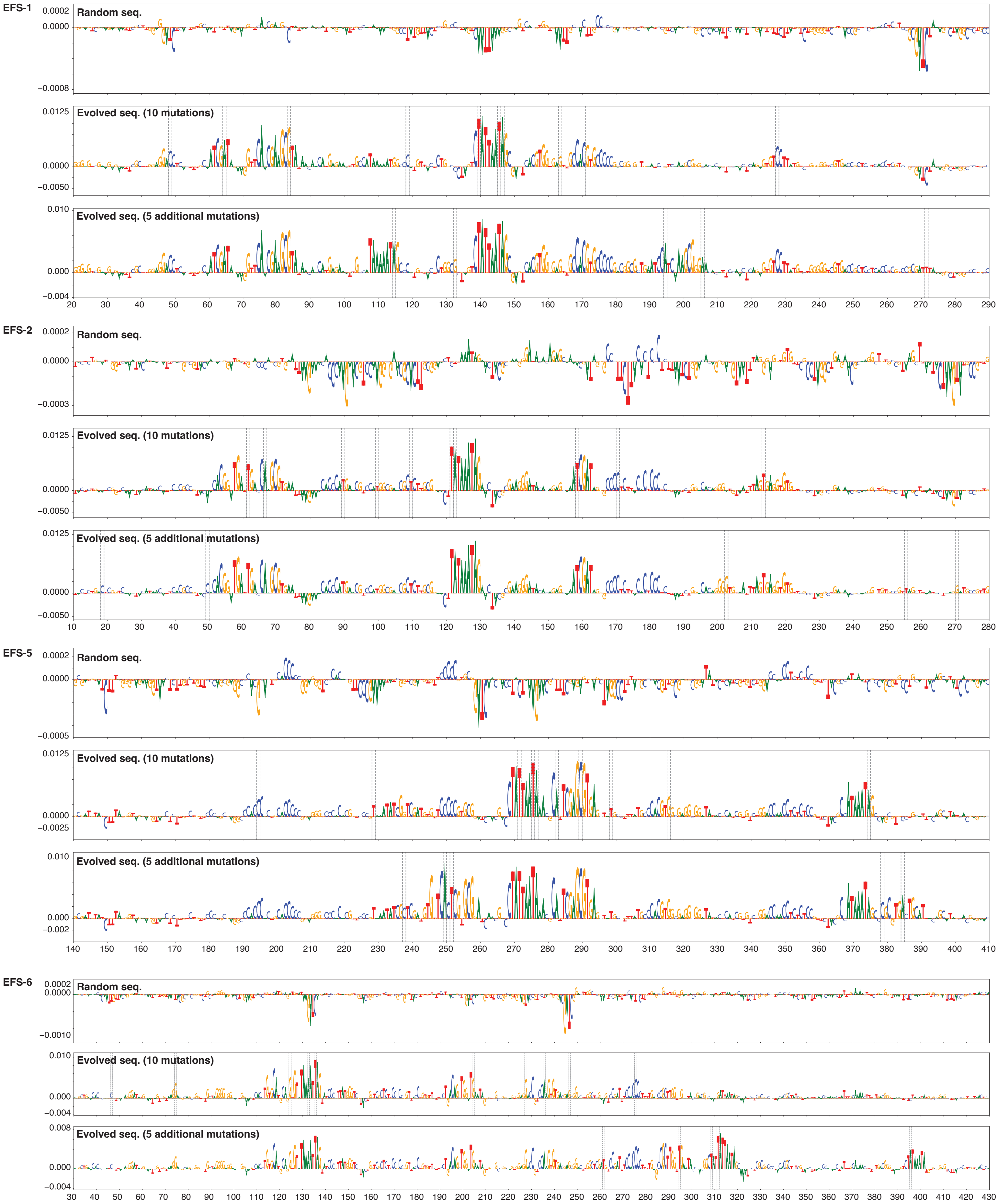


**Supplementary Figure 3: Kenyon cell in silico evolution, 5 additional mutations (EFS-1, EFS-2, EFS-5, EFS-6)**
Nucleotide contribution score (DeepFlyBrain KC class) of the original random sequence (top) and evolved sequence after 10 mutations (middle) and 15 mutations (bottom) for each selected in silico evolution sequence. Location of mutated nucleotides is indicated by dashed lines.

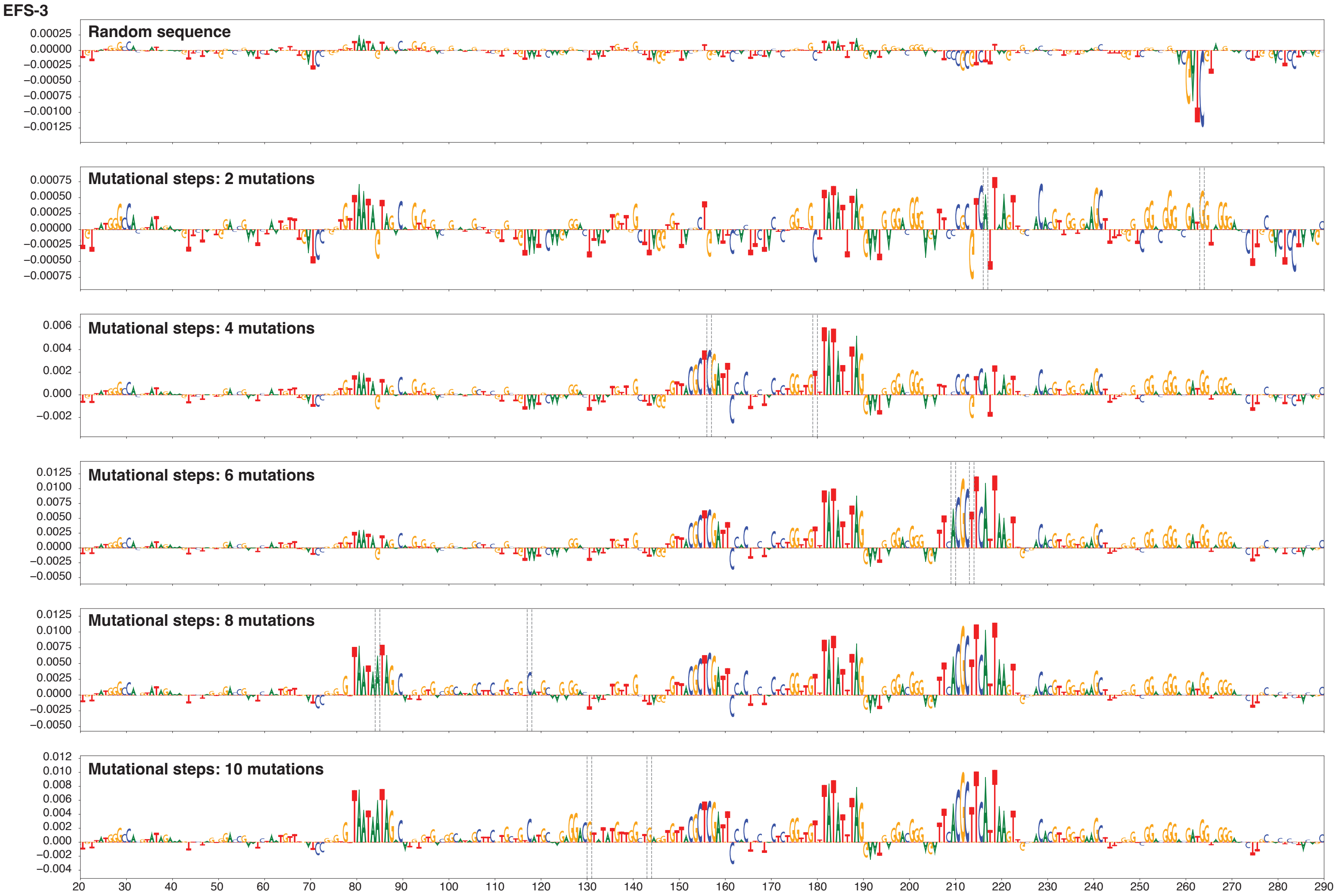


**Supplementary Figure 4: Kenyon cell in silico evolution 2 - 4 - 6 - 8 - 10 mutations (EFS-3)**
Nucleotide contribution score (DeepFlyBrain KC class) of the original random sequence (top) and evolved sequence at every two steps of the in silico evolution process. Location of mutated nucleotides is indicated by dashed lines.

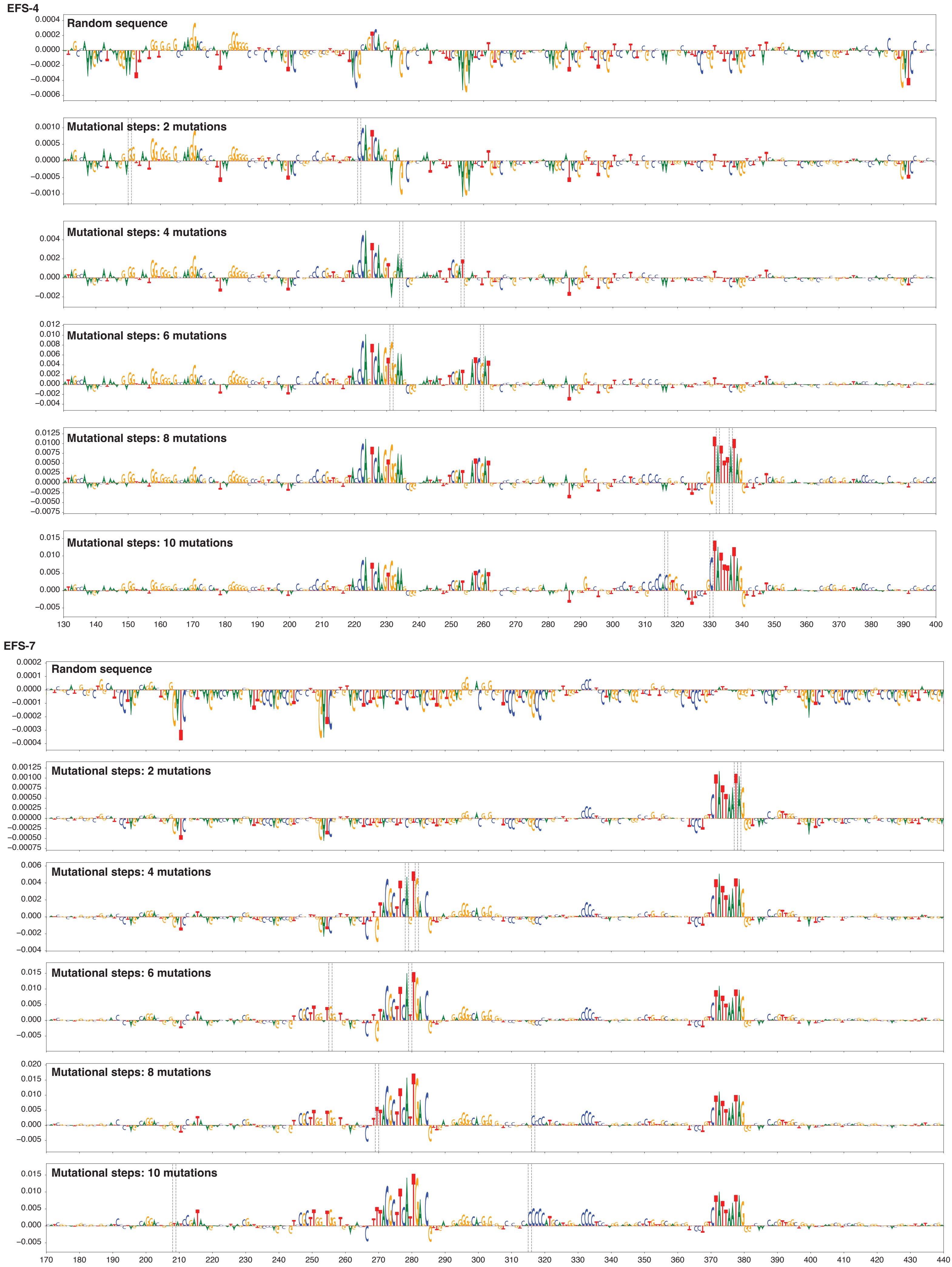


**Supplementary Figure 5: Kenyon cell in silico evolution 2 - 4 - 6 - 8 - 10 mutations (EFS-4, EFS-7)**
Nucleotide contribution score (DeepFlyBrain KC class) of the original random sequence (top) and evolved sequence at every two steps of the in silico evolution process. Location of mutated nucleotides is indicated by dashed lines.

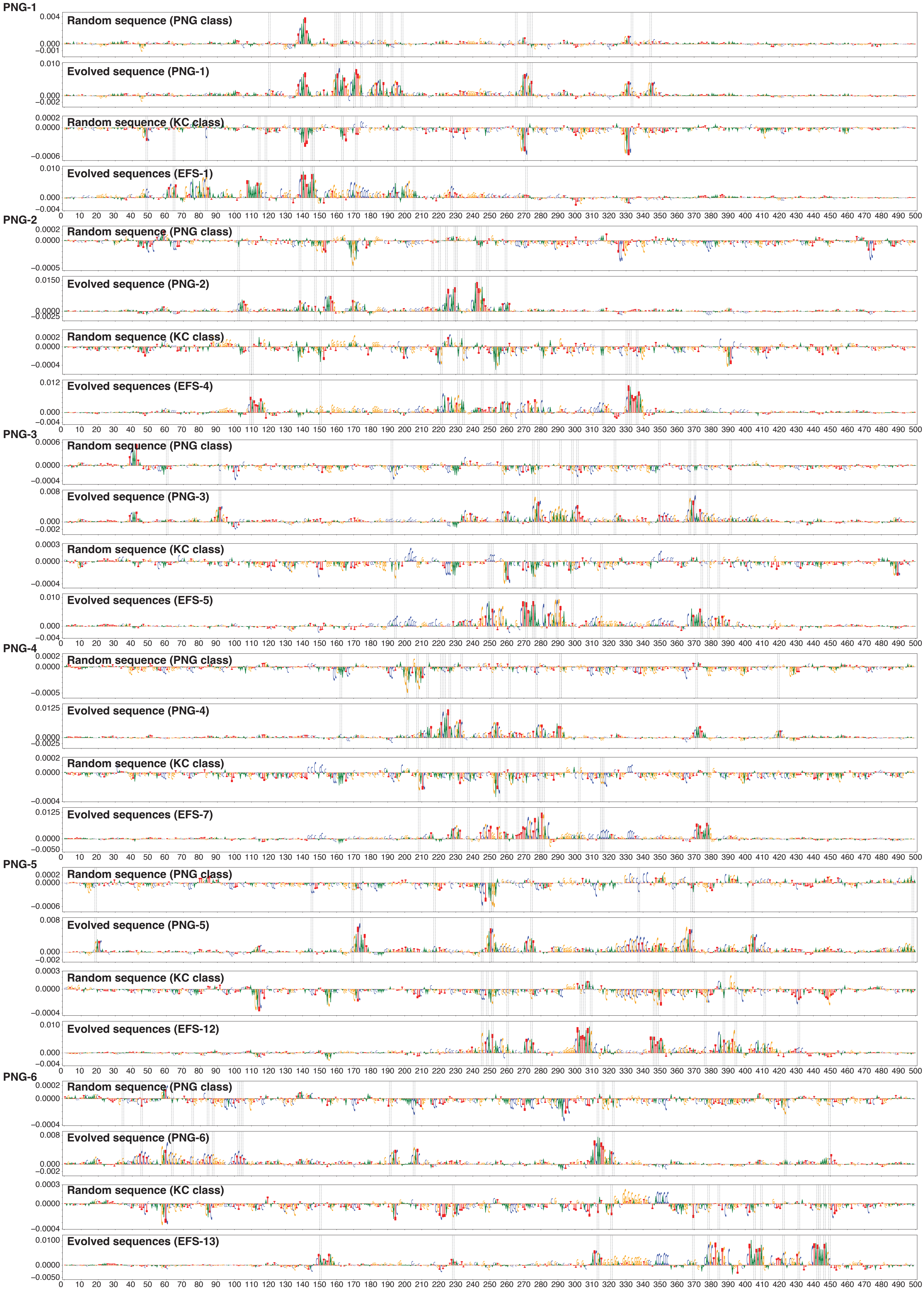


**Supplementary Figure 6: Perineurial glia in silico evolution**

Nucleotide contribution score (DeepFlyBrain) of the original random (1st-3rd) and evolved PNG (2nd) and KC (4th) sequences.

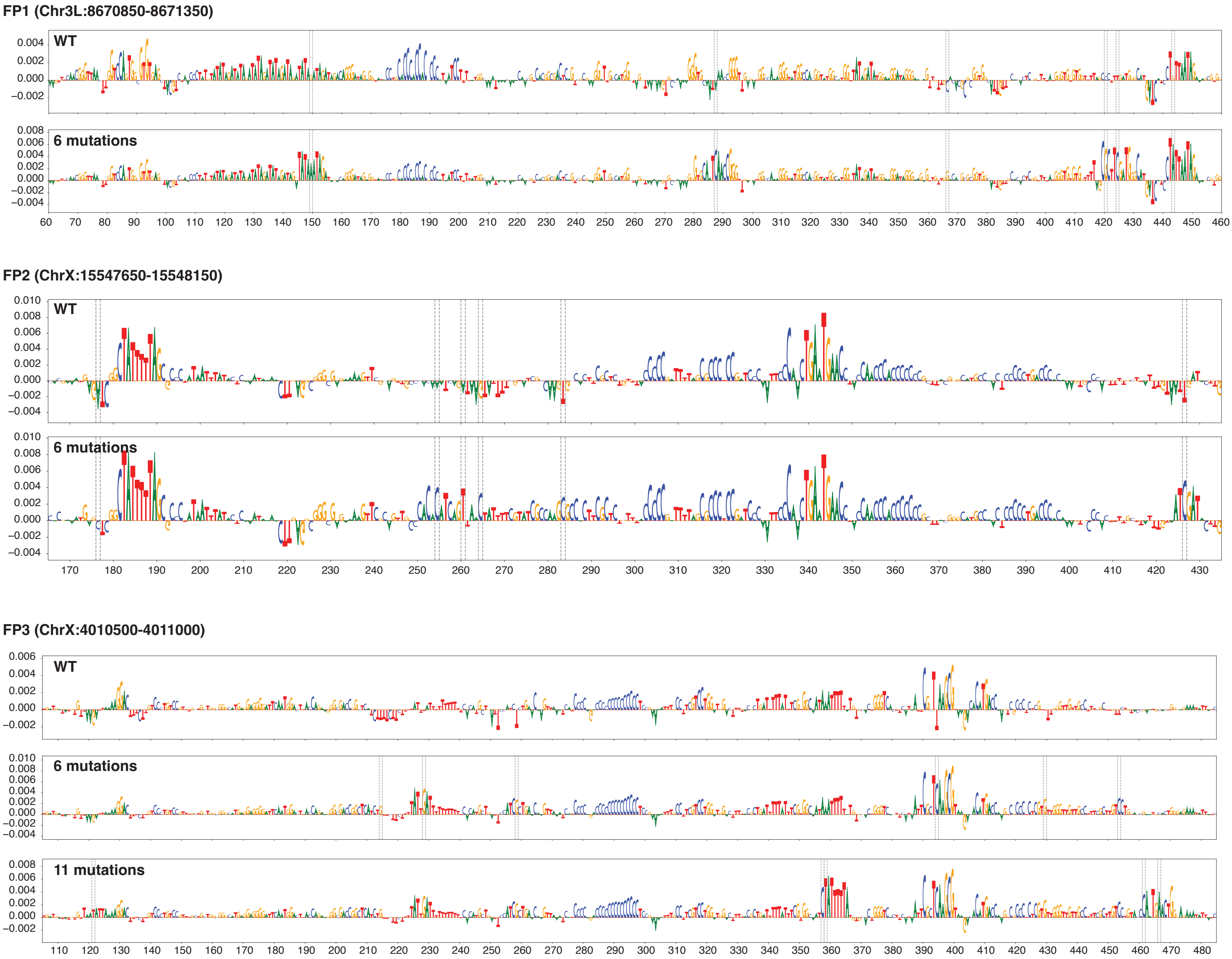


**Supplementary Figure 7: Kenyon cell in silico evolution, “Near enhancers” rescue**
Nucleotide contribution score (DeepFlyBrain KC class) of the original genomic sequence (top) and evolved sequence (bottom) for each “near enhancer” sequence. Location of mutated nucleotides is indicated by dashed lines.

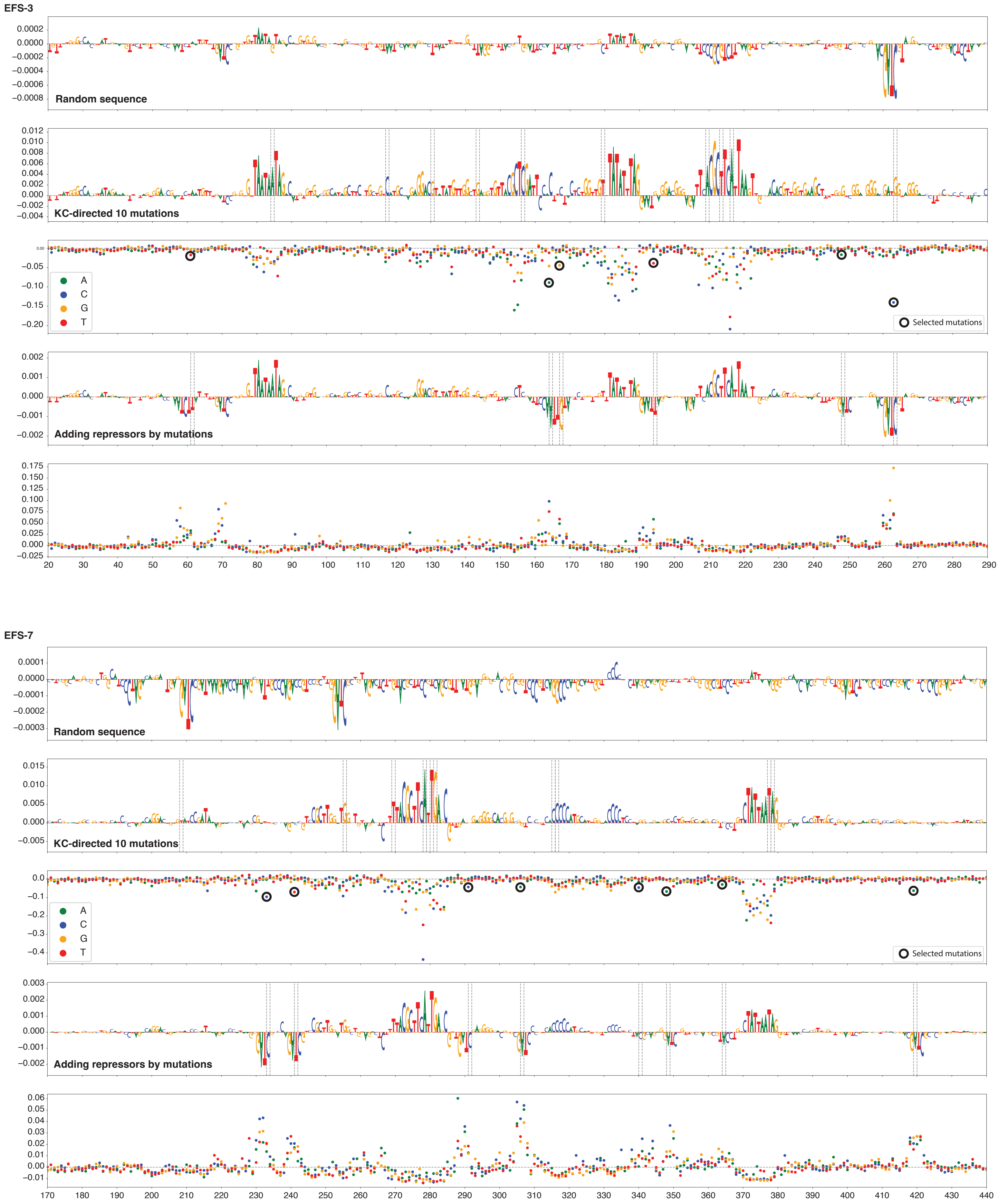


**Supplementary Figure 8: Kenyon cell in silico evolution, addiyion of repressor sites**
Nucleotide contribution score (DeepFlyBrain KC class) of the original random sequence (1st), evolved sequence (2nd), and after generating repressor sites (4th) for each EFS-3 and EFS-7sequence. Delta prediction score for in silico saturation mutagenesis of the evolved sequence (3rd) and after addition of repressors sites (5th). Location of mutated nucleotides is indicated by dashed lines.

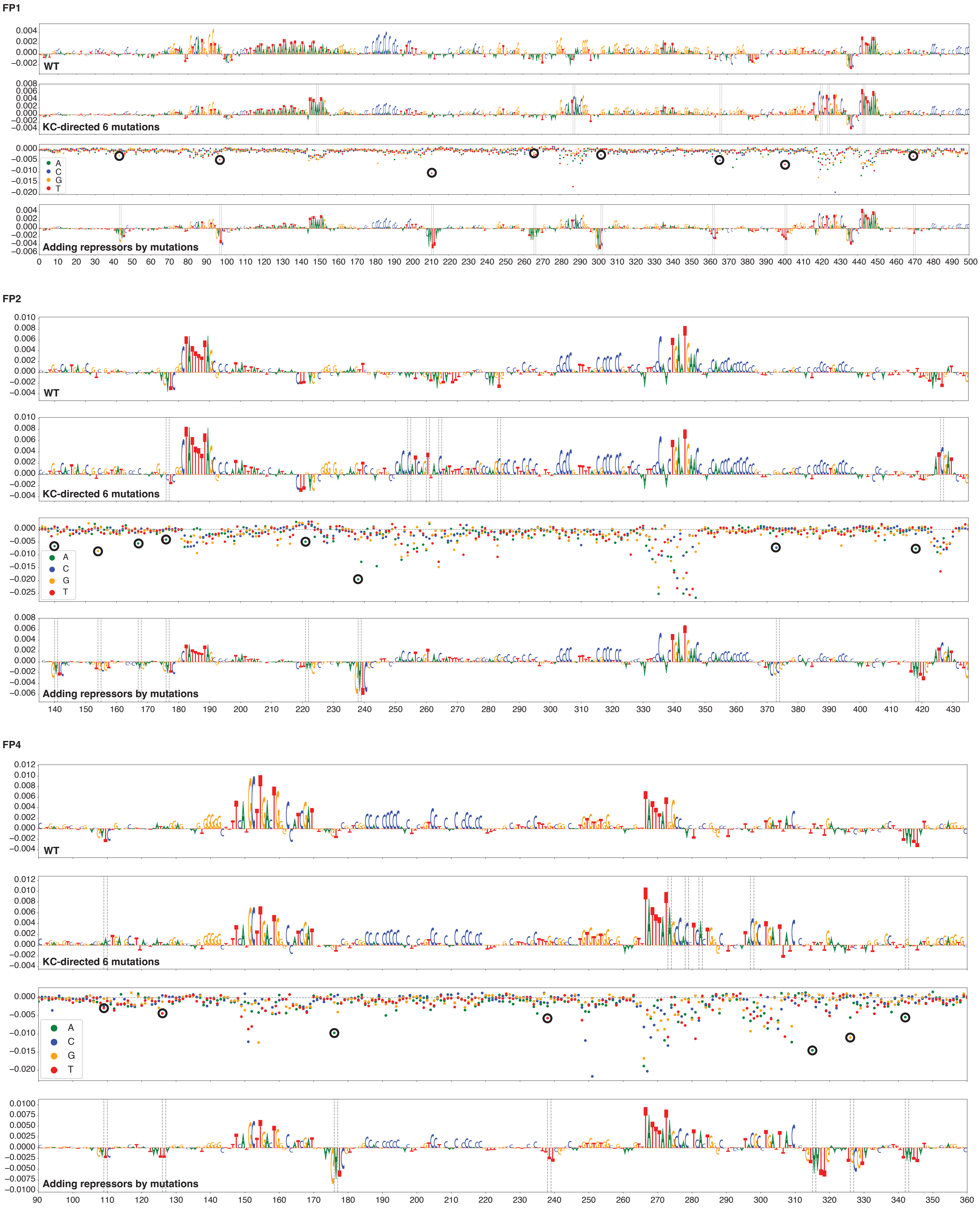


**Supplementary Figure 9: Kenyon cell in silico evolution, "Near enhancers" addition of repressor sites**
Nucleotide contribution score (DeepFlyBrain KC class) of the original random sequence (1st), evolved sequence (2nd), and after generating repressor sites (4th) for each "near enhancer" sequence. Delta prediction score for in silico saturation mutagenesis of the evolved sequence (3rd). Location of mutated nucleotides is indicated by dashed lines.

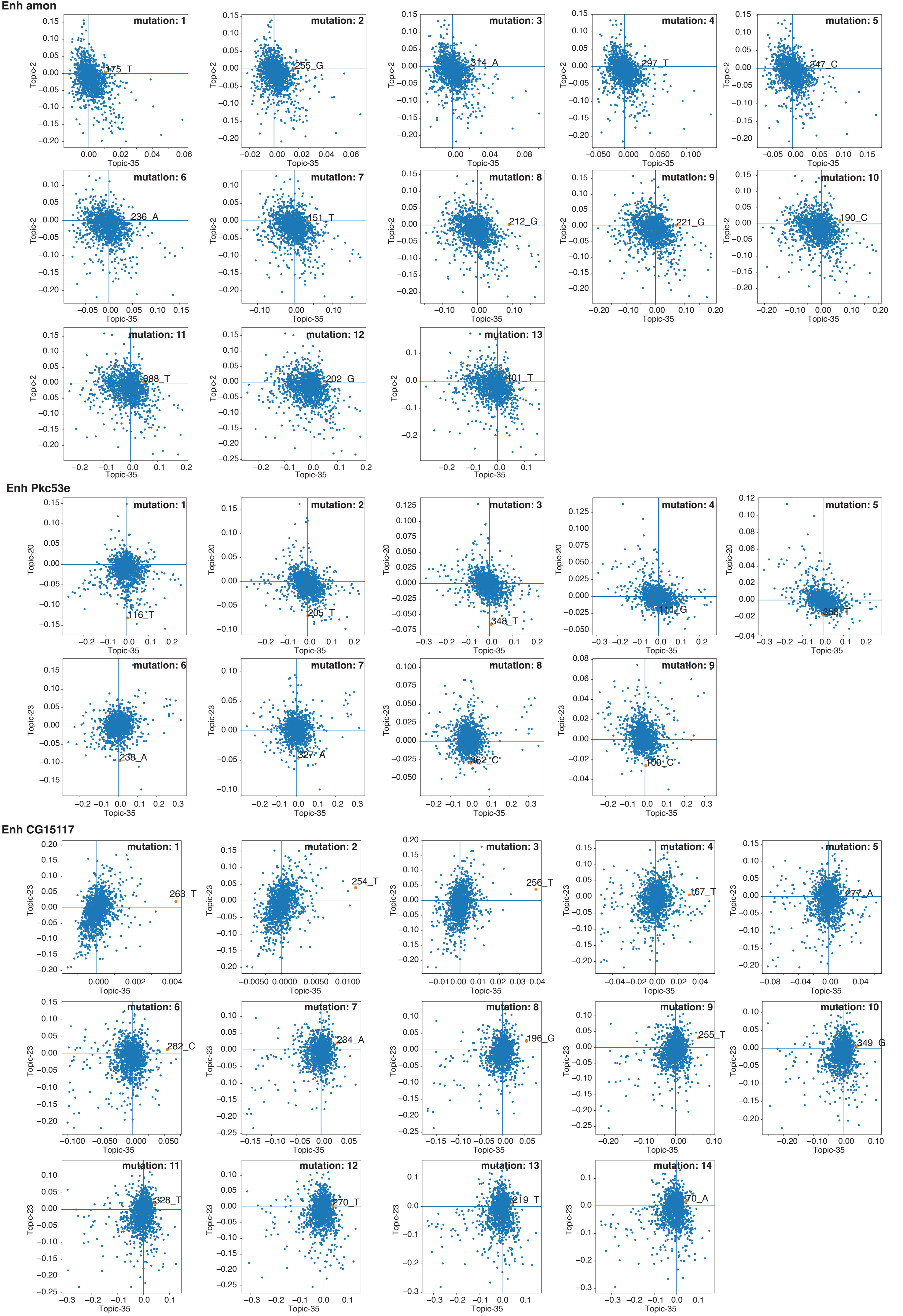


**Supplementary Figure 10: Dual code enhancer in silico evolution, Mutation selection**

In silico saturation mutagenesis prediction scores for each selected mutation. Topic 2: T4; Topic 20: T2; Topic 23: T1; Topic 35: KC.

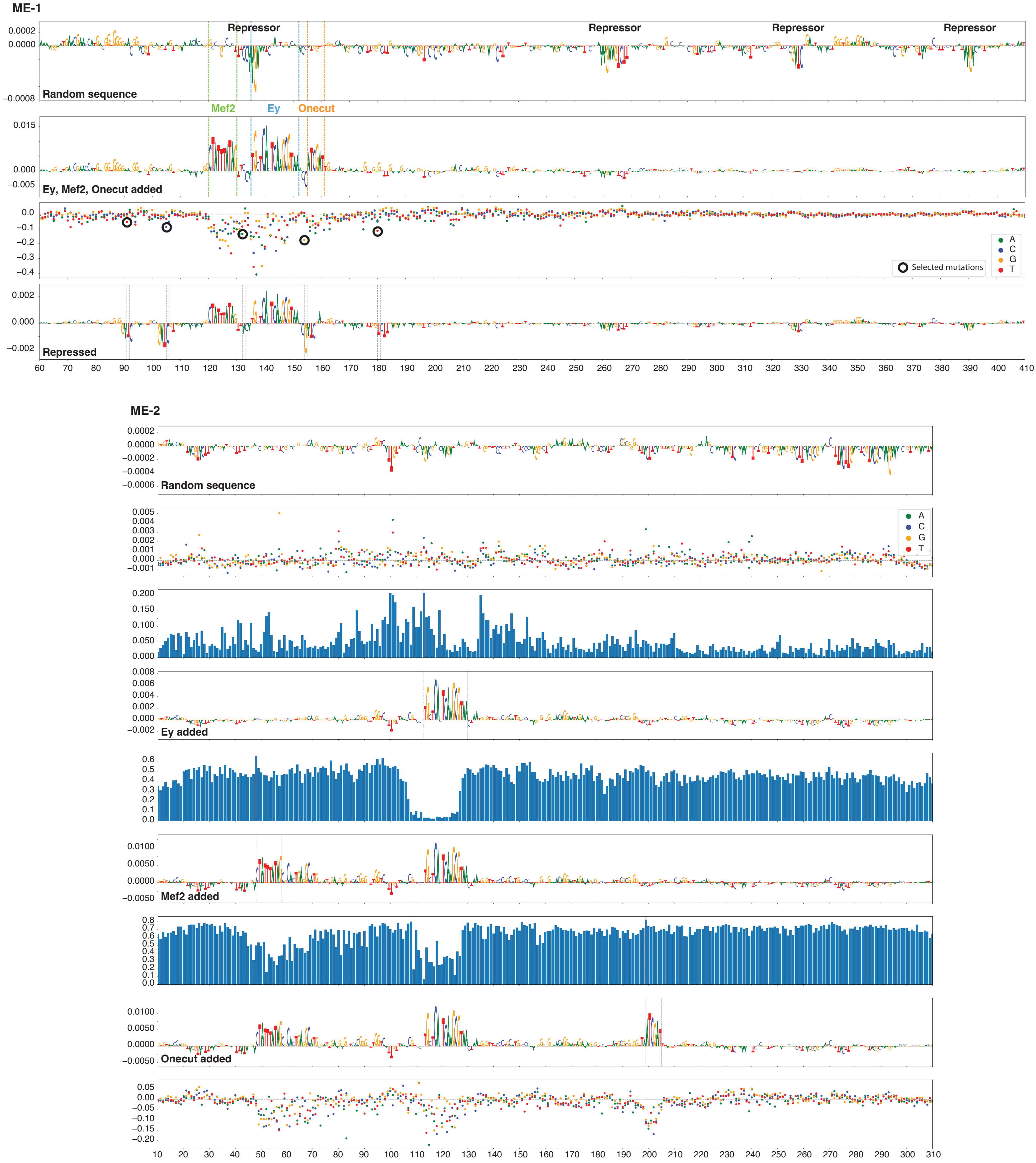


**Supplementary Figure 11: Kenyon cell motif implanting**

**ME-1:** Nucleotide contribution score (DeepFlyBrain KC class) of the original random sequence (1st), after motif implanting (2nd), and after generation of repressor sites (4th). In silico saturation mutagenesis after motif implanting (3rd). Location of motifs and selected mutations are indicated by dashed lines. **ME-2:** Nucleotide contribution score (DeepFlyBrain KC class) of the original random sequence (1st) and after each motif addition (4th, 6th, 8th). In silico saturation mutagenesis of the original random sequence (2nd) after motif implanting (9th). Delta prediction score of motif implanted over the whole enhancer for Ey (3rd), Mef2 (5th), and Onecut (7th). Selected implanting location is indicated by a red dashed line. Implanted motifs are indicated by grey dashed lines.

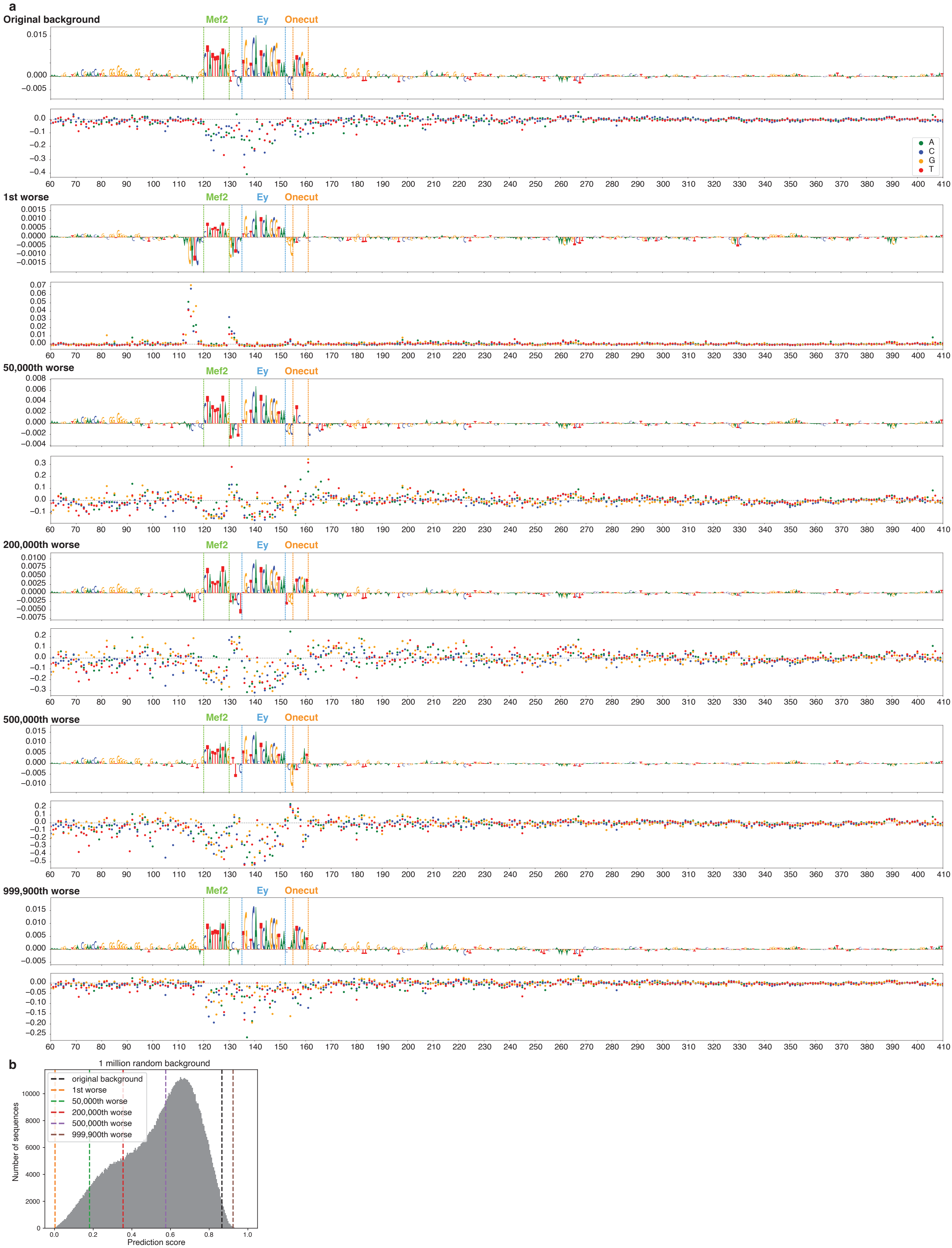


**Supplementary Figure 12: Kenyon cell motif implanting, variation of background sequence in short enhancer**

**a**, Nucleotide contribution score (top) and delta prediction score for in silico saturation mutagenesis (bottom) of the original background and the 1st, 50,000th, 200,000th, 500,000th, and 999,900th worse background. Mef2, Ey, and Onecut motif location is indicated in green, blue and orange respectively. **b**, Distribution of prediction score for the 49 bp short enhancer with 1,000,000 random background. Dashed lines indicate prediction scores for the original background (black) and the 1st (orange), 50,000th (green), 200,000th (red), 500,000th (purple), and 999,900th (brown) worse background.

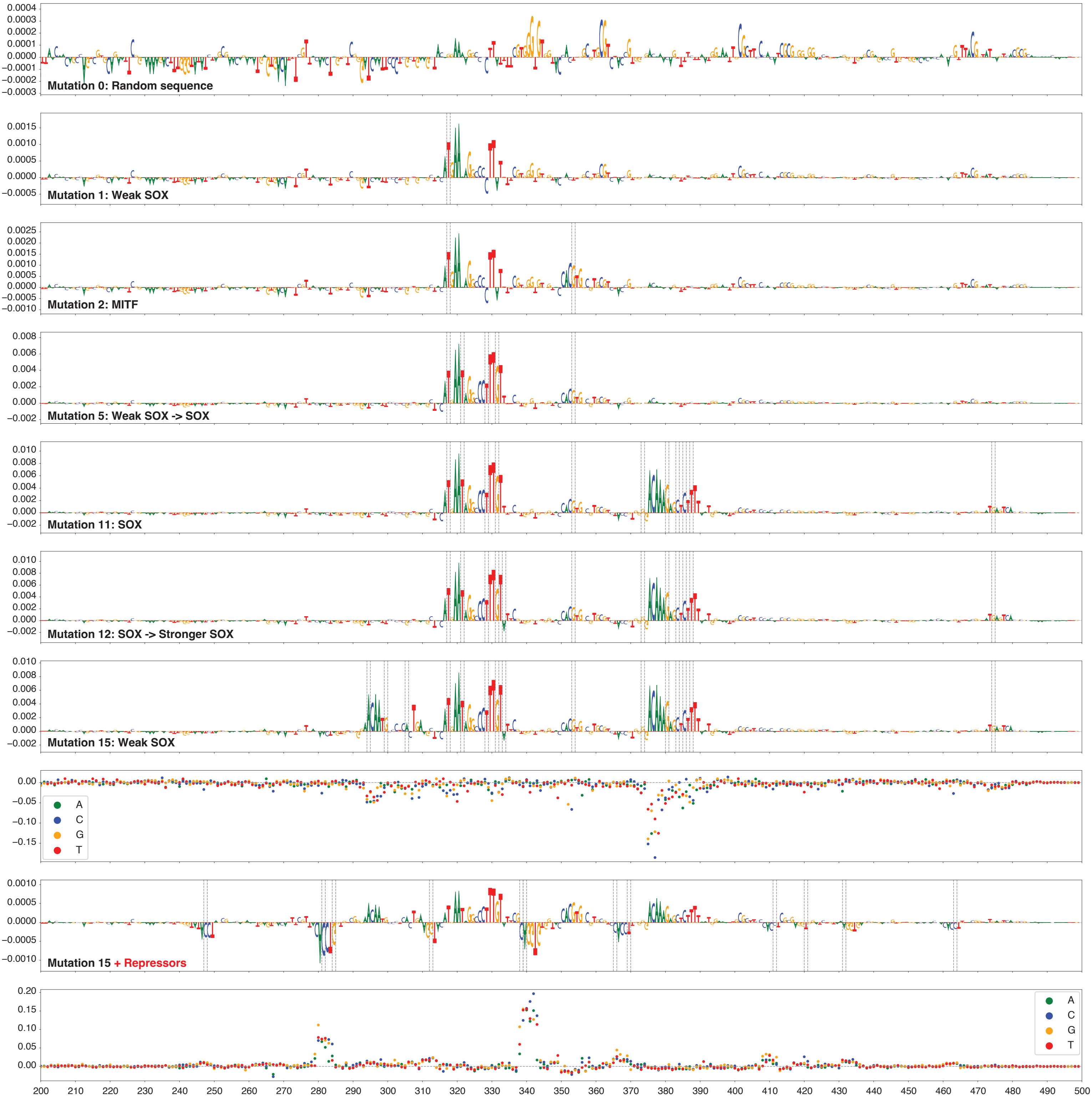


**Supplementary Figure 13: MEL melanoma in silico evolution (EFS-1 all variants)**
Nucleotide contribution score (DeepMEL2 MEL class) of the original random sequence (1st), after 1, 2, 5 , 11, 12, and 15 mutations (2nd - 7th), and after generation of repressor sites (9th). Delta prediction score for in silico saturation mutagenesis of the evolved sequence (8th) and after generation of repressor sites (10th). Location of mutations is indicated by dashed lines.

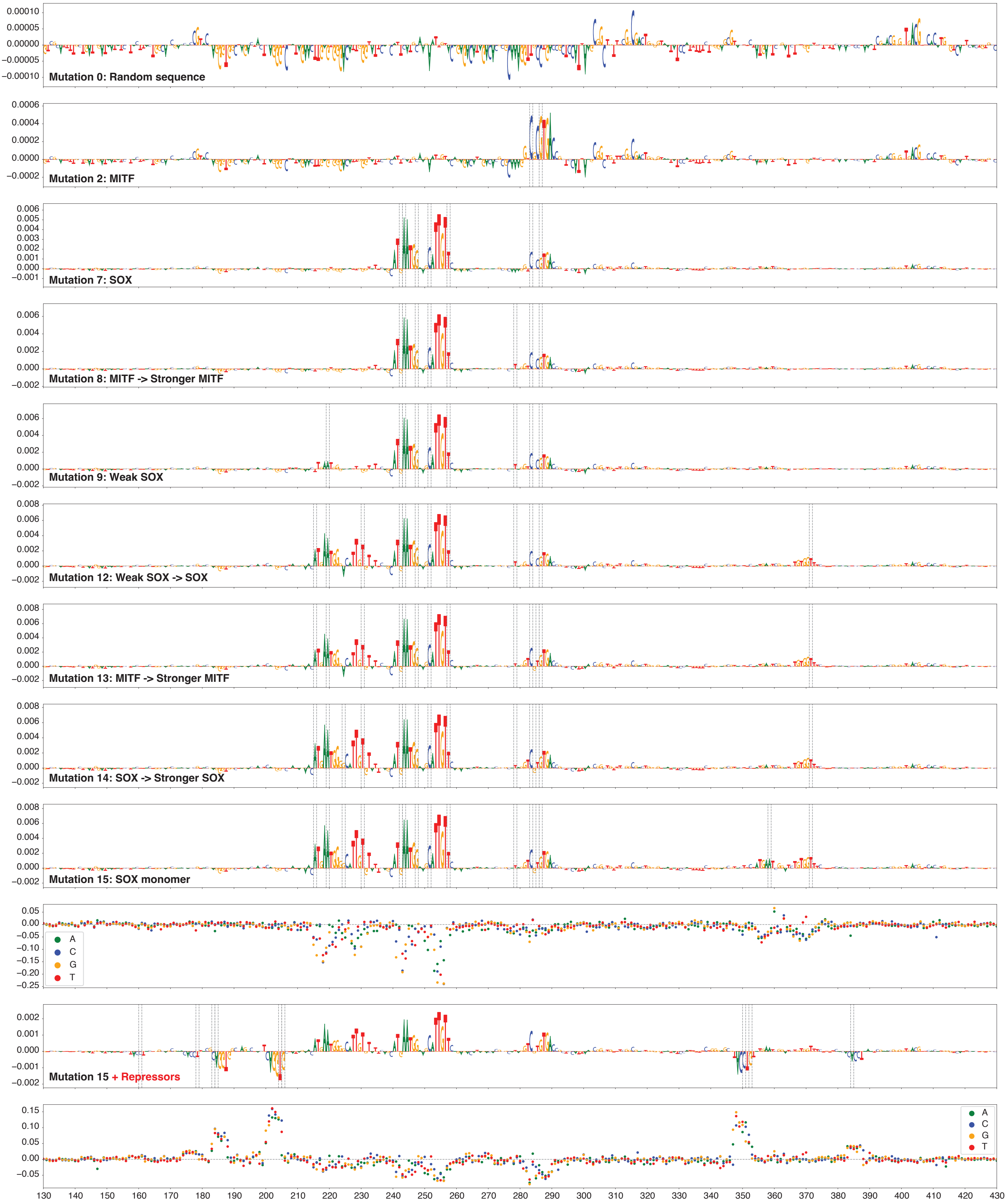


**Supplementary Figure 14: MEL melanoma in silico evolution (EFS-8 all variants)**
Nucleotide contribution score (DeepMEL2 MEL class) of the original random sequence (1st), after 2, 7, 8 , 9, 12, 13, 14 and 15 mutations (2nd - 9th), and after generation of repressor sites (11th). Delta prediction score for in silico saturation mutagenesis of the evolved sequence (10th) and after generation of repressor sites (12th). Location of mutations is indicated by dashed lines.

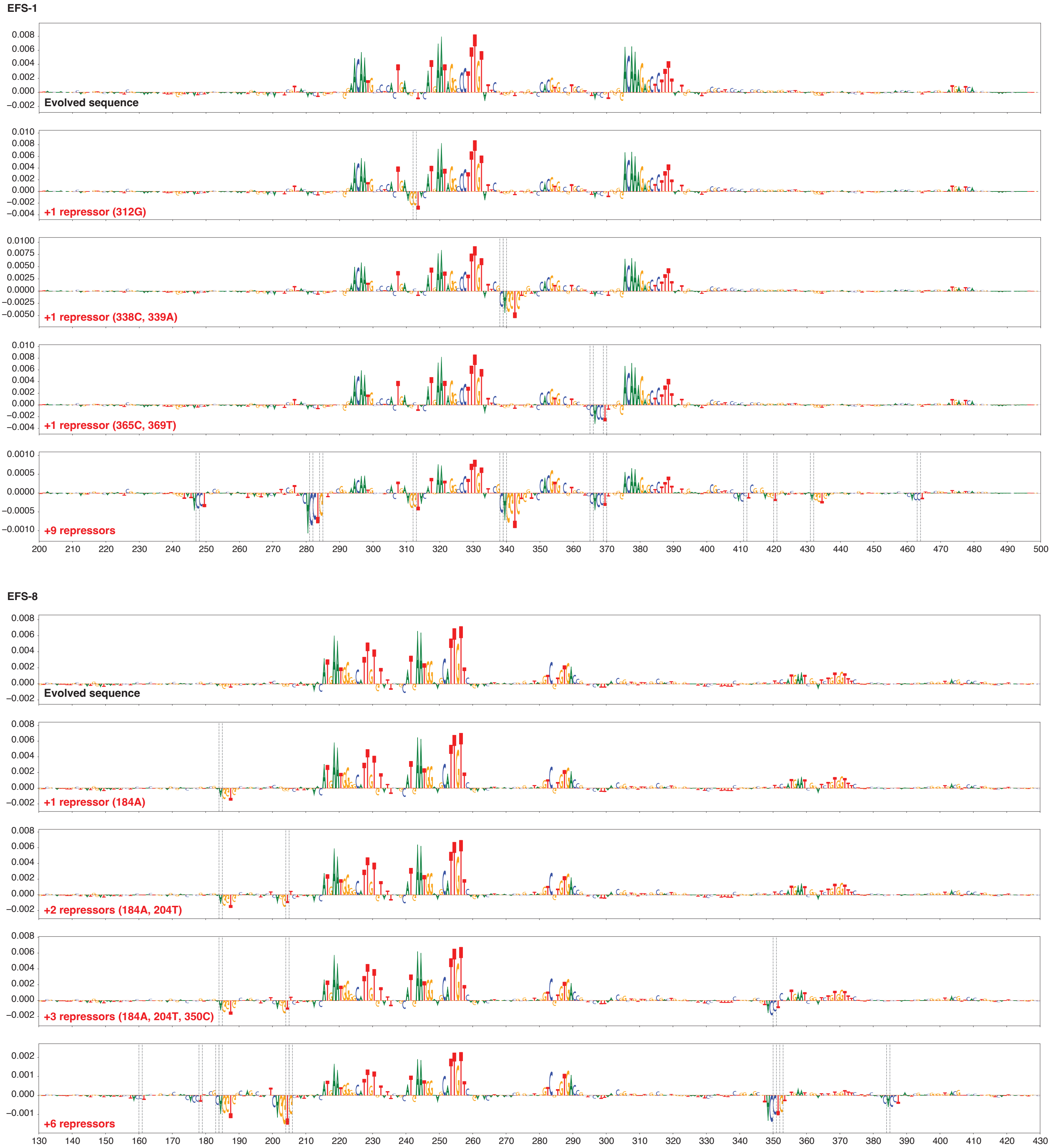


**Supplementary Figure 15: MEL melanoma in silico evolution, EFS-1 and EFS-8 consecutive repressor addition**
Nucleotide contribution score (DeepMEL2 MEL class) of the evolved sequence (1st) and after mutations generating one or multiple repressor sites. Location of mutations is indicated by dashed lines.

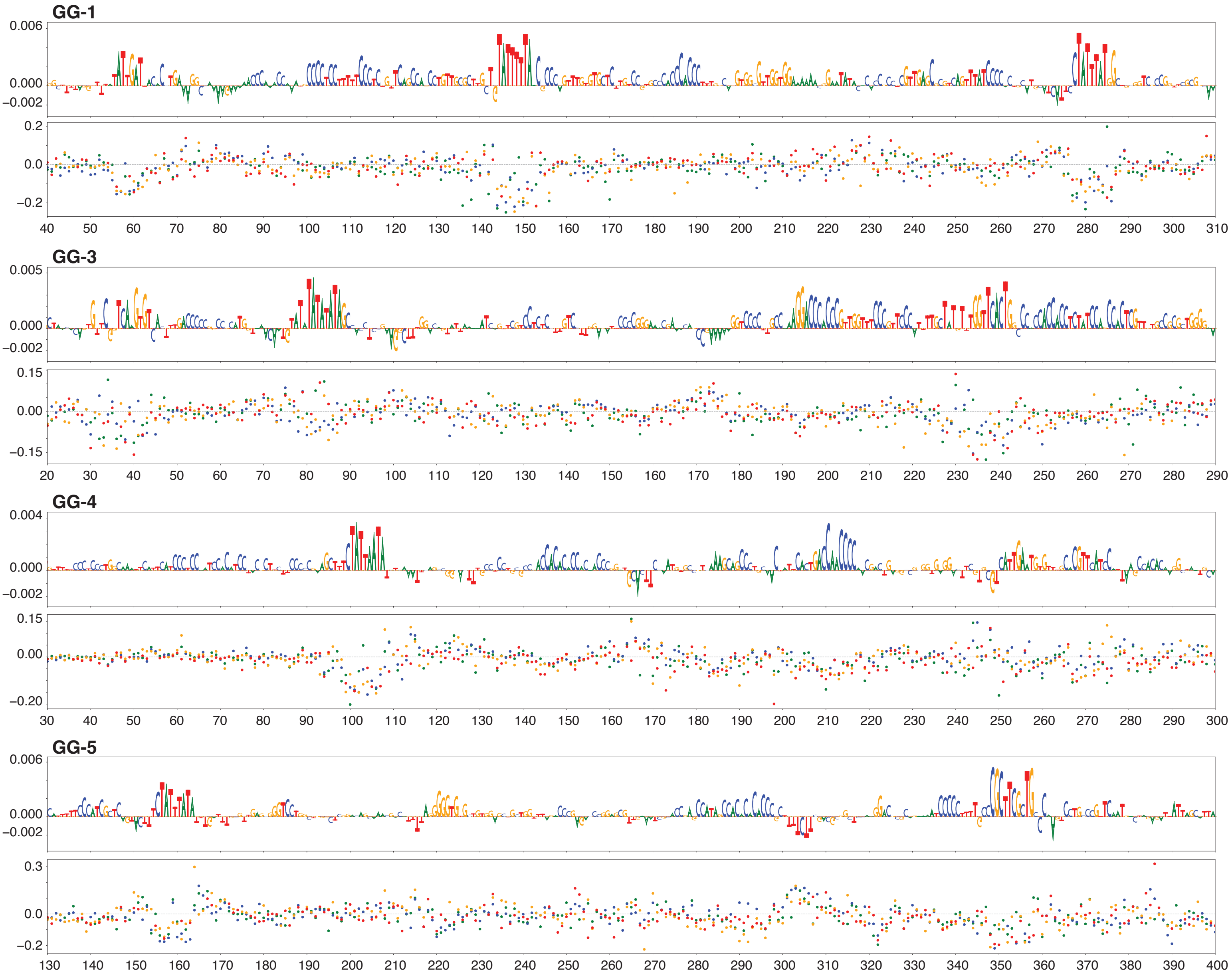


**Supplementary Figure 16: Kenyon cell GAN enhancers**
Nucleotide contribution score (DeepFlyBrain KC class) of the GAN generated sequence (top). Delta prediction score for in silico saturation mutagenesis (bottom).
