# Supplementary material for: Cell-type-directed design of synthetic enhancers
Source: Nature. 2023 Dec 12;626(7997):212–20. doi: 10.1038/s41586-023-06936-2 (PMC10830415; doi:10.1038/s41586-023-06936-2)
Supplement: Supplementary file 2 — Reporting Summary [file 41586_2023_6936_MOESM2_ESM.pdf]

nature portfolio

Corresponding author(s): Stein Aerts

Last updated by author(s): Nov 22, 2023

Reporting Summary

Nature Portfolio wishes to improve the reproducibility of the work that we publish. This form provides structure for consistency and transparency in reporting. For further information on Nature Portfolio policies, see our Editorial Policies and the Editorial Policy Checklist.

Statistics

For all statistical analyses, confirm that the following items are present in the figure legend, table legend, main text, or Methods section.

n/a	Confirmed	
☐	☒	The exact sample size (*n*) for each experimental group/condition, given as a discrete number and unit of measurement
☒	☐	A statement on whether measurements were taken from distinct samples or whether the same sample was measured repeatedly
☒	☐	The statistical test(s) used AND whether they are one- or two-sided<br>*Only common tests should be described solely by name; describe more complex techniques in the Methods section.*
☒	☐	A description of all covariates tested
☒	☐	A description of any assumptions or corrections, such as tests of normality and adjustment for multiple comparisons
☐	☒	A full description of the statistical parameters including central tendency (e.g. means) or other basic estimates (e.g. regression coefficient) AND variation (e.g. standard deviation) or associated estimates of uncertainty (e.g. confidence intervals)
☒	☐	For null hypothesis testing, the test statistic (e.g. *F*, *t*, *r*) with confidence intervals, effect sizes, degrees of freedom and *P* value noted<br>*Give P values as exact values whenever suitable.*
☒	☐	For Bayesian analysis, information on the choice of priors and Markov chain Monte Carlo settings
☒	☐	For hierarchical and complex designs, identification of the appropriate level for tests and full reporting of outcomes
☐	☒	Estimates of effect sizes (e.g. Cohen's *d*, Pearson's *r*), indicating how they were calculated

Our web collection on statistics for biologists contains articles on many of the points above.

Software and code

Policy information about availability of computer code

Data collection	Confocal images: ZEN blue 3.4.91
Data analysis	Custom codes: https://doi.org/10.5281/zenodo.10184648<br><br>DL Python environment to use DeepMEL 1.0, DeepMEL2 1.0, and DeepFlyBrain 1.0:<br>python=3.7 tensorflow-gpu=1.15 numpy=1.19.5 matplotlib=3.1.1 shap=0.29.3 ipykernel=5.1.2 h5py=2.10.0<br><br>DL Python environment to train GAN models:<br>python=3.6 tensorflow-gpu=1.14.0 keras-gpu=2.2.4 numpy=1.16.2 matplotlib=3.1.1 shap=0.29.3 ipykernel=5.1.2<br><br>To perform motif analysis: TF-Modisco 0.5.5.4, Tomtom (MEME 5.5.1), ClusterBuster 2022-04-21, BEDTools 2.30.0<br>To create higher-order background sequences: INCLUSive 3.2<br><br>To calculate statics: Scipy 1.6.0<br><br>To train ChromBPNet model: ChromBPNet 1.3-pre-release<br><br>ATAC-seq and ChIP-seq data analysis:<br>Demultiplexing with bcl2fastq 2.20<br>Adapter trimming with trimgalore 0.6.7<br>Mapping with bwa-mem2 2.2.1

Sorting with SAMtools 1.16.1
Deduplicating with SAMtools 1.16.1
Removing blacklist regions with SAMtools 1.16.1
Generating bigwig with deepTools 3.5.0
Peak calling with MACS2 2.1.2.1

For manuscripts utilizing custom algorithms or software that are central to the research but not yet described in published literature, software must be made available to editors and reviewers. We strongly encourage code deposition in a community repository (e.g. GitHub). See the Nature Portfolio guidelines for submitting code & software for further information.

Data

Policy information about availability of data

All manuscripts must include a data availability statement. This statement should provide the following information, where applicable:

- Accession codes, unique identifiers, or web links for publicly available datasets
- A description of any restrictions on data availability
- For clinical datasets or third party data, please ensure that the statement adheres to our policy

Cloned Drosophila and human sequences were provided as Supplementary Tables. DeepMEL, DeepMEL2, and DeepFlyBrain deep learning model files were obtained from Kipoi (http://kipoi.org/models/DeepMEL, https://kipoi.org/models/DeepFlyBrain) with Zenodo record ids 3592129, 4590308, and 5153337. The fasta files used to train GAN models and the trained GAN models are available on Zenodo at https://doi.org/10.5281/zenodo.6701504. Custom genomes (hg38 and dm6) generated in this study are available on Zenodo at https://doi.org/10.5281/zenodo.10184648. Chromatin accessibility values in Kenyon Cells in adult Drosophila brains were obtained from GSE16369739. In vitro saturation mutagenesis on IRF4 data was obtained from https://kircherlab.bihealth.org/satMutMPRA/. Chromatin accessibility of Drosophila and transduced melanoma lines and ZEB2 ChIP-seq data generated for this study have been submitted to the NCBI Gene Expression Omnibus (GEO, https://www.ncbi.nlm.nih.gov/geo/) under accession number GSE240003.

Research involving human participants, their data, or biological material

Policy information about studies with human participants or human data. See also policy information about sex, gender (identity/presentation), and sexual orientation and race, ethnicity and racism.

Reporting on sex and gender	No human research participants involved
Reporting on race, ethnicity, or other socially relevant groupings	No human research participants involved
Population characteristics	No human research participants involved
Recruitment	No human research participants involved
Ethics oversight	No human research participants involved

Note that full information on the approval of the study protocol must also be provided in the manuscript.

Field-specific reporting

Please select the one below that is the best fit for your research. If you are not sure, read the appropriate sections before making your selection.

☒ Life sciences ☐ Behavioural & social sciences ☐ Ecological, evolutionary & environmental sciences

For a reference copy of the document with all sections, see nature.com/documents/nr-reporting-summary-flat.pdf

Life sciences study design

All studies must disclose on these points even when the disclosure is negative.

Sample size	The number of synthetic enhancers that were tested using transgenic flies was determined to be minimally 6 per cell type and it was bounded by the feasibility of the transgenic animal generation experiments. In total, 68 transgenic flies were generated.<br>The number of synthetic enhancers that were used with luciferase assays is determined to be minimally 10 per different category (in silico evolution, motif embedding, GAN, repressors, mutational steps). In total, 97 sequences were tested using luciferase assay.
Data exclusions	No data was excluded.
Replication	The same results were obtained from different replication experiments.<br>Multiple brains (at least 10) were stained and imaged for the fly experiments.<br>3 biological replicates were performed for the main luciferase experiments.<br>2 biological replicates were performed for the negative control luciferase experiments.<br>No biological replicates perfomed on ATAC-seq or ChIP-seq experiments.
Randomization	The initial random sequences (used for sequence evolution and motif implantation) were sampled from the sequence space that matches the

Randomization	GC content of the genomic sequences.<br>Flies fitting the gender(equal amount of male and female) and age (<10days) criteria were selected randomly for all experiments.<br>In this study, we didn't perform experiments that needed to be allocated into different groups.
Blinding	The investigators were blinded when performing cloning, transfection, antibody staining, and luciferase experiments by using enhancer IDs.

Reporting for specific materials, systems and methods

We require information from authors about some types of materials, experimental systems and methods used in many studies. Here, indicate whether each material, system or method listed is relevant to your study. If you are not sure if a list item applies to your research, read the appropriate section before selecting a response.

Materials & experimental systems

n/a	Involved in the study
☐	☒ Antibodies
☐	☒ Eukaryotic cell lines
☒	☐ Palaeontology and archaeology
☐	☒ Animals and other organisms
☒	☐ Clinical data
☒	☐ Dual use research of concern
☒	☐ Plants

Methods

n/a	Involved in the study
☐	☒ ChIP-seq
☒	☐ Flow cytometry
☒	☐ MRI-based neuroimaging

Antibodies

Antibodies used	1 - Rabbit polyclonal anti-GFP (1:1000 dilution); Life Technologies CAT# A-6455; RRID: AB_221570<br>2 - Donkey polyclonal anti-rabbit Alexa Fluor 488 (1:500 dilution); Life Technologies CAT# A-21206; RRID: AB_2535792<br>3 - Rabbit anti-ZEB2; Bethyl CAT# A302-473A (1mg/ml and we used 5 micrograms for ChIP)<br>4 - Mouse anti-Dachshund (1:250 dilution); DSHB; CAT# dac1-1<br>5 - Alexa Fluor 647 goat anti-mouse IgG (1:500 dilution); Invitrogen, CAT# A-21235
Validation	1- References provided, statement on manufacturer's website: "This Antibody was verified by Relative expression to ensure that the antibody binds to the antigen stated.". Selected references out of 238: PMID 36067320, 35142344, 34908527, 34644579, 33932333, 33846330, 33463521, 33174166, 33112231, 32640222.<br>2- References provided, no statement on manufacturer's website. Selected references out of 6277: PMID 36067320, 35142344, 34908527, 34644579, 33932333, 33846330, 33463521, 33174166, 33112231, 32640222.<br>3- Testing and references provided, we performed ChIP-seq using ZEB2 antibody and the most enriched motif was the ZEB2 motif. No statement on the manufacturer's website. References: PMID 33614228, 20515682<br>4- References provided, statement on manufacturer's website: "The antibody reproduces the pattern observed by in situ hybridization with a dac cDNA probe (unpublished observations) and an enhancer trap insert in dac.". References: PMID 7821215, 17868668, 32781577, 18430931, 25670791, 8756723, 9845371, 24142104, 22874913, 34409041, 34322481, 33982759, 32738261, 32781577, 32184260, 31453329.<br>5- References provided, statement on the manufacturer's website: "The antibody "was used with a concentration of 2µg/mL.". Selected references out of 1448: PMID 35297981, 35017509, 33570489, 32878938, 32649914, 33659324, 32579612, 32317641, 37332603, 36879821, 36355348, 36649336, 34459871, 34605405, 33689682.

Eukaryotic cell lines

Policy information about cell lines and Sex and Gender in Research

Cell line source(s)	MM001, MM047, and MM099 were obtained from Prof. Dr. Ghanem Ghanem with a Material Transfer Agreement.<br>HEK293T was obtained from ATCC (CAT# CRL-3216).
Authentication	We have used MM001, MM047, and MM099 in previous studies (Verfaillie et al., Nature Communications, 2015; Wouters et al., Nature Cell Biology 2020; Minnoye et al., Genome Research, 2020; Kalender-Atak et al., Genome Research 2021). We authenticated the cell lines by tracking their morphology overtime and by checking their genomic profile and mutations (Verfaillie et al., Kalender-Atak et al.), transcriptomic profile (Wouters et al.), and epigenomic profile (Verfaillie et al., Wouters et al., Kalender-Atak et al.).<br>HEK293T cells were only used for lentivirus production in this study, and the final products were tested and confirmed by sequencing. No authentication was needed for this cell line.
Mycoplasma contamination	Cell lines were tested for mycoplasma contamination, and were found negative.
Commonly misidentified lines<br>(See ICLAC register)	No commonly misidentified cell lines were used in this study.

Animals and other research organisms

Policy information about studies involving animals; ARRIVE guidelines recommended for reporting animal research, and Sex and Gender in Research

Laboratory animals	Transgenic Drosophila melanogaster strains were used in this study. Young adult flies (<10-days-old) were used when performing antibody stainings.
Wild animals	No wild animals were used.
Reporting on sex	Sexes were equally mixed when performing antibody staining on adult Drosophila melanogaster brains.
Field-collected samples	No field-collected samples were used.
Ethics oversight	No approval required.

Note that full information on the approval of the study protocol must also be provided in the manuscript.

Plants

Seed stocks	No plants were used.
Novel plant genotypes	No plants were used.
Authentication	No plants were used.

ChIP-seq

Data deposition

☒ Confirm that both raw and final processed data have been deposited in a public database such as GEO.

☒ Confirm that you have deposited or provided access to graph files (e.g. BED files) for the called peaks.

Data access links<br>*May remain private before publication.*	GSE240003
Files in database submission	MM001_ZEB2_ChIP-seq<br>MM001_input_ChIP-seq
Genome browser session<br>(e.g. UCSC)	no longer applicable

Methodology

Replicates	n=1
Sequencing depth	ZEB2_ChIP-seq: 83410868<br>input_ChIP-seq: 168512695
Antibodies	Rabbit anti-ZEB2; Bethyl CAT# A302-473A
Peak calling parameters	macs2 callpeak default parameters
Data quality	31866 peaks are called with 5% FDR
Software	Demultiplexing with bcl2fastq 2.20<br>adapter trimming with trimgalore 0.6.7<br>mapping with bwa-mem2 2.2.1<br>sorting with SAMtools 1.16.1<br>deduplicating with SAMtools 1.16.1<br>removing blacklist regions with SAMtools 1.16.1<br>generating bigwig with deepTools 3.5.0

peak calling with MACS2 2.1.2.1
