# Supplementary material for: Cell-type-directed design of synthetic enhancers
Source: Nature. 2023 Dec 12;626(7997):212–20. doi: 10.1038/s41586-023-06936-2 (PMC10830415; doi:10.1038/s41586-023-06936-2)
Supplement: Supplementary file 4 — All scripts used to design sequences, analyse data and reproduce figures. [file 41586_2023_6936_MOESM4_ESM.zip › Taskiran_et_al_Supplemental_Code/General/Sequence_evolution.html]

Sequence\_evolution


### The following script was run using a conda environment. Below you can find how to create the same environment¶

In [ ]:

```
# conda create -n deeplearning_py37_tf115 python=3.7
# conda activate deeplearning_py37_tf115
# pip install tensorflow-gpu==1.15
# conda install cudnn
# conda install -c conda-forge shap
# conda install matplotlib
# conda install ipykernel
# conda install numpy==1.19.5
# conda install h5py==2.10.0
```

### Load utils and necessary functions¶

In [1]:

```
print('Loading utils and functions...')

import tensorflow as tf
tf.disable_eager_execution()
tf.logging.set_verbosity(tf.logging.ERROR)
import utils
import numpy as np
import matplotlib.pyplot as plt
%matplotlib inline
```

```
Loading utils and functions...
```

### Load the data¶

##### (to be used to initialize DeepExplainer)¶

In [2]:

```
import pickle
print('Loading data...')
f = open('./data/DeepFlyBrain_data.pkl', "rb")
DeepFlyBrain_data_dict = pickle.load(f)
f.close()
```

```
Loading data...
```

### Load the model¶

In [3]:

```
print('Loading model...')

name = "DeepFlyBrain"
model_json_file = "./models/DeepFlyBrain/DeepFlyBrain_architecture.json"
model_hdf5_file = "./models/DeepFlyBrain/DeepFlyBrain_weights.hdf5"

model_dict = {}
model_dict[name] = utils.load_model(model_json_file, model_hdf5_file)
```

```
Loading model...
```

### Create random sequences¶

In [4]:

```
number_of_random_regions = 10
seq_len = 500


insilico_evolution_dict = {}
insilico_evolution_dict["regions"] = utils.random_sequence(seq_len, number_of_random_regions)
```

### (or) Create random sequences by shuffling a given sequence.¶

##### (Length of the given sequence should be the same size you used for the model, which is 500bp in this case)¶

In [5]:

```
number_of_random_regions = 10
seq_to_shuffle = "AGGGTCGGGCGTGTCCGCCTGTTGGAATATGCTTCTCAGGTCTTCTGGGAAACAGATGTTTTGTGGAAGTGGAAGATTTTGGAAGTAGTGCCTTATCATGTGAAACCACAGGGCAGCTGATCTCTTCAGGCTTTCTTGATGTGAATGACAGCTTTGTTTCATCCACTTTGGTGGGTAAAAGAAGGCAAATTCCCCTGTGGTACTTTTGGTGCCAGGTTTAGCCATATGACGAAGCTTTACATAAAACAGTACAAGTATCTCCATTGTCCTTTATGATCCTCCATGAGTGTTTTCACTTAGTCTGATGAAGGGTTCACTCCAGTCTTTTCGGATGATAAAATGCTTCGGCTGTCAGTCTAATAAGGGATTCCCTGAGGAGTTTGGAGGCTGTAAGAGCACCCCCCGTCTCAATGCCAGTGCTTCTTATCTCAGCCTCTCCTGCACTCCTTTACCCCCGTCTCGATGCCAGTGCTTCCTATCTCAGCCTCTCCTGCACTCCT"


insilico_evolution_dict = {}
insilico_evolution_dict["regions"] = utils.random_sequence_by_shuffling(seq_to_shuffle, number_of_random_regions)
```

### (or) Create random sequences that follow the GC-content of the given fasta sequences¶

In [6]:

```
path_to_use_GC_content = "./data/KC_regions.fa"
number_of_random_regions = 10
seq_len = 500


insilico_evolution_dict = {}
insilico_evolution_dict["regions"] = utils.random_sequence_gc_adjusted(seq_len, number_of_random_regions, path_to_use_GC_content)
```

### Here you generate synthetic sequence by in silico evolution.¶

##### **region:** initial regions to be evolved (random or genomic)¶

##### **model:** selected deep learning model¶

##### **class\_no:** chosen class to direct the sequence evolution¶

##### **n\_mutation:** number of iterative mutations to perform¶

##### As an output **mutation\_pred** contains the prediction scores after each mutation and **mutation\_loc** contains the location and substitute of each mutation¶

In [7]:

```
path_to_save = "./output/testdata_sequence_evolution_10seqs.pkl"

insilico_evolution_dict["mutation_pred"], \
insilico_evolution_dict["mutation_loc"] = utils.insilico_evolution(regions = insilico_evolution_dict["regions"],
                                                                   model = model_dict["DeepFlyBrain"],
                                                                   class_no = 35,
                                                                   n_mutation = 10)
# save the final file
import pickle
f = open(path_to_save, "wb")
pickle.dump(insilico_evolution_dict,f)
f.close()
```

```
Sequence index: 0,1,2,3,4,5,6,7,8,9,
```

### Load the saved file¶

In [8]:

```
path_to_file = "./output/testdata_sequence_evolution_10seqs.pkl"


import pickle
f = open(path_to_file, "rb")
insilico_evolution_dict = pickle.load(f)
f.close()
```

### Plot the prediction score change (each sequence as a line)¶

In [9]:

```
class_no = 35

_ = plt.plot(insilico_evolution_dict["mutation_pred"][:,:,class_no-1].T,alpha=0.2)
plt.xlabel("Number of Mutations")
plt.ylabel("Prediction Score")
```

Out[9]:

```
Text(0, 0.5, 'Prediction Score')
```

### Plot the prediction score distribution after each mutation¶

In [10]:

```
class_no = 35
n_mutation = 10

_ = plt.boxplot(insilico_evolution_dict["mutation_pred"][:,:n_mutation+1,class_no-1],notch=True,showfliers=False, whis=[5,95])
_ = plt.xticks(range(1,n_mutation+2),list(range(n_mutation+1)))
plt.xlabel("Number of Mutations")
plt.ylabel("Prediction Score Distribution")
```

Out[10]:

```
Text(0, 0.5, 'Prediction Score Distribution')
```

### Plot prediction of a sequence at different steps¶

In [11]:

```
index = 2
class_no = 35

plt.bar(range(insilico_evolution_dict["mutation_pred"][index,:,class_no-1].shape[0]),insilico_evolution_dict["mutation_pred"][index,:,class_no-1])
plt.xlabel("Number of mutations")
plt.ylabel("Prediction score")
```

Out[11]:

```
Text(0, 0.5, 'Prediction score')
```

### Plot deepexplainer and saturation mutagenesis of the selected step¶

In [12]:

```
index = 2
step = 5
class_no =  35

# Initialize the explainer for the selected class
import shap
tf.disable_eager_execution()
rn=np.random.choice(DeepFlyBrain_data_dict["nonAugmented_data"].shape[0], 250, replace=False)
name = "DeepFlyBrain"
explainer_dict = {} 
explainer_dict[name+"_c"+str(class_no)] = shap.DeepExplainer((model_dict[name].inputs,
                                                              model_dict[name].layers[-1].output[:,class_no-1]),
                                                             DeepFlyBrain_data_dict["nonAugmented_data"][rn])

# Apply the selected mutations on the initial random sequence
nuc_to_onehot = {"A":[1, 0, 0, 0],"C":[0, 1, 0, 0],"G":[0, 0, 1, 0],"T":[0, 0, 0, 1]}
onehot_ = np.copy(insilico_evolution_dict["regions"][index:index+1])
for i, mut_ in enumerate(insilico_evolution_dict["mutation_loc"][index][:step]):
    print(mut_,end=",")
    onehot_[0][int(mut_.split("_")[0]),:] = np.array(nuc_to_onehot[mut_.split("_")[1]], dtype='int8')
print("") 


# Plot contribution scores and saturation mutagenesis
ntrack = 2
fig = plt.figure(figsize=(80,ntrack*5))
ax1 = utils.plot_deepexplainer_givenax(explainer=explainer_dict["DeepFlyBrain_c35"], fig=fig, ntrack=ntrack, track_no=1, seq_onehot=onehot_)
_ = utils.plot_mutagenesis_givenax(model=model_dict["DeepFlyBrain"], fig=fig, ntrack=ntrack, track_no=2, seq_onehot=onehot_, class_no = class_no)
```

```
387_A,409_C,406_G,416_G,342_C,
```

#### Convert created sequence to letters¶

In [13]:

```
index = 2
step = 5

nuc_to_onehot = {"A":[1, 0, 0, 0],"C":[0, 1, 0, 0],"G":[0, 0, 1, 0],"T":[0, 0, 0, 1]}
onehot_ = np.copy(insilico_evolution_dict["regions"][index:index+1])

print("Performed mutations:",end=" ")
for i, mut_ in enumerate(insilico_evolution_dict["mutation_loc"][index][:step]):
    print(mut_,end=",")
    onehot_[0][int(mut_.split("_")[0]),:] = np.array(nuc_to_onehot[mut_.split("_")[1]], dtype='int8')   
print("")

print("Generated sequence:",end=" ")
for nuc in onehot_[0]:
    if nuc[0]==1:
        print("A",end="")
    if nuc[1]==1:
        print("C",end="")
    if nuc[2]==1:
        print("G",end="")
    if nuc[3]==1:
        print("T",end="")
print("")
```

```
Performed mutations: 387_A,409_C,406_G,416_G,342_C,
Generated sequence: CCTATAGTTCTGTAAATATTGGGGCCGTCTTCTAATTTATCTAGCAATCCGAGCCATGAAGCTACGGTGTACTTACAGCCGAATTGGTCCTCTGCGGATTTAACGTCAATTTAACATAATGAACGTTGTTGTCATTGCGCTACACAATTTTTAAATCATTCCCTACCCAGTCTGCCGCCGAGATGTGAAAAGTGGTAACTCGAGCGTCAAGGGAGCTTAGCGTTGCCATGATTAGGATTCTGAAGCCTATCTTGTGACTAAGATCCCCCCAAAGGCCGTAGCGGGACTTACGCATCAGCCCTTTGAGCTGCCATTTAATAGCGTCGCTGTCAGATGATTGAACAGCCGGAACATACGTCTGTATGTCGCGAATGCTTGGGCGAGTTCATCGCTATGAGTAAAATATGAGCATTGACGCGTGAATAAGCAGCGGATATTTCCGACGCCGGGACATAAAGCGTTACCTTGACGTGCTACTTACCGATATCACCCTCTCCGGA
```

In [ ]:

```

```
