# Supplementary material for: Cell-type-directed design of synthetic enhancers
Source: Nature. 2023 Dec 12;626(7997):212–20. doi: 10.1038/s41586-023-06936-2 (PMC10830415; doi:10.1038/s41586-023-06936-2)
Supplement: Supplementary file 4 — All scripts used to design sequences, analyse data and reproduce figures. [file 41586_2023_6936_MOESM4_ESM.zip › Taskiran_et_al_Supplemental_Code/General/Generative_model.html]

Generative\_model


### The GAN models were trained using a conda environment. Below you can find how to create the same environment to train GAN models and generate sequences¶

In [ ]:

```
# %%bash
# conda create --name deeplearning_py36_tf114_gpu python=3.6 tensorflow-gpu=1.14.0 keras-gpu=2.2.4
# conda activate deeplearning_py36_tf114_gpu
# conda install numpy=1.16.2  matplotlib=3.1.1 shap=0.29.3 ipykernel=5.1.2
```

### Below you can find explanation of parameters that were used to train model and generate sequences.¶

#### (These variables can be found in the beginning of **wgan\_gp.py**)¶

**BATCH\_SIZE**: Batch size (how many regions will be used in each iteration). \
**ITERS**: Number of batch iterations to train the model. \
**SEQ\_LEN**: Length of the input sequences. \
**SEQ\_DIM**: Dimension of the input sequences. (4 nucleotides) \
**DIM**: Dimension of the model. It is used in latent space and convolutional layers. \
**CRITIC\_ITERS**: How many training iterations will be done for Discriminator for each Generator iteration. \
**LAMBDA**: Hyperparameter for gradient penalty. \
**loginterval**: Once every N iteration the log will be saved. \
**seqinterval**: Once every N iteration the sample sequences will be generated. \
**modelinterval**: Once every N iteration the model files will be saved. \
**selectedmodel**: When generating sequences, the iteration number of the model you want to use. \
**suffix**: When generating sequences, the suffix to add to the header of the fasta regions. \
**ngenerate**: When generating sequences, number of sequences you want to generate relative to batchsize. Example: 1 (128 sequences will be generated if the batch size is 128) \
**outputdirc**: Path the to output folder. \
**fastafile**: Path to the fasta file to use as real enhancers

### How to run the model¶

In [ ]:

```
# %%bash
# conda activate deeplearning_py36_tf114_gpu
# python wgan_gp.py
```

### This will result following outputs¶

**./models/**: Folder containing saved model's weight files. \
**./samples\_ACGT/**: Folder containing sampled sequences during training. \
**./samples\_raw/**: Folder containing sampled sequences (in their raw format) during training. \
**./gen\_seq/**: Folder containing generated sequences after training. \
**./disc.json**: Architecture file of the discriminator. \
**./gen.json**: Architecture file of the generator. \
**./d\_g\_loss.pkl**: Logged loss values during training.

In [ ]:

```

```
