# Supplementary material for: Cell-type-directed design of synthetic enhancers
Source: Nature. 2023 Dec 12;626(7997):212–20. doi: 10.1038/s41586-023-06936-2 (PMC10830415; doi:10.1038/s41586-023-06936-2)
Supplement: Supplementary file 4 — All scripts used to design sequences, analyse data and reproduce figures. [file 41586_2023_6936_MOESM4_ESM.zip › Taskiran_et_al_Supplemental_Code/General/Motif_implantation.html]

Motif\_implantation


### The following script was run using a conda environment. Below you can find how to create the same environment¶

In [ ]:

```
# conda create -n deeplearning_py37_tf115 python=3.7
# conda activate deeplearning_py37_tf115
# pip install tensorflow-gpu==1.15
# conda install cudnn
# conda install -c conda-forge shap
# conda install matplotlib
# conda install ipykernel
# conda install numpy==1.19.5
# conda install h5py==2.10.0
```

### Load utils and necessary functions¶

In [1]:

```
print('Loading utils and functions...')

import tensorflow as tf
tf.disable_eager_execution()
tf.logging.set_verbosity(tf.logging.ERROR)
import utils
import numpy as np
import matplotlib.pyplot as plt
%matplotlib inline
```

```
Loading utils and functions...
```

### Load the data¶

##### (to be used to initialize DeepExplainer)¶

In [2]:

```
import pickle
print('Loading data...')
f = open('./data/DeepFlyBrain_data.pkl', "rb")
DeepFlyBrain_data_dict = pickle.load(f)
f.close()
```

```
Loading data...
```

### Load the model¶

In [3]:

```
print('Loading model...')

name = "DeepFlyBrain"
model_json_file = "./models/DeepFlyBrain/DeepFlyBrain_architecture.json"
model_hdf5_file = "./models/DeepFlyBrain/DeepFlyBrain_weights.hdf5"

model_dict = {}
model_dict[name] = utils.load_model(model_json_file, model_hdf5_file)
```

```
Loading model...
```

### Create random sequences¶

In [4]:

```
number_of_random_regions = 100
seq_len = 500


motif_embedding_dict = {}
motif_embedding_dict["Random"] = {}
motif_embedding_dict["Random"]["regions"] = utils.random_sequence(seq_len, number_of_random_regions)
```

### (or) Create random sequences by shuffling a given sequence.¶

##### (Length of the given sequence should be the same size you used for the model, which is 500bp in this case)¶

In [5]:

```
number_of_random_regions = 100
seq_to_shuffle = "AGGGTCGGGCGTGTCCGCCTGTTGGAATATGCTTCTCAGGTCTTCTGGGAAACAGATGTTTTGTGGAAGTGGAAGATTTTGGAAGTAGTGCCTTATCATGTGAAACCACAGGGCAGCTGATCTCTTCAGGCTTTCTTGATGTGAATGACAGCTTTGTTTCATCCACTTTGGTGGGTAAAAGAAGGCAAATTCCCCTGTGGTACTTTTGGTGCCAGGTTTAGCCATATGACGAAGCTTTACATAAAACAGTACAAGTATCTCCATTGTCCTTTATGATCCTCCATGAGTGTTTTCACTTAGTCTGATGAAGGGTTCACTCCAGTCTTTTCGGATGATAAAATGCTTCGGCTGTCAGTCTAATAAGGGATTCCCTGAGGAGTTTGGAGGCTGTAAGAGCACCCCCCGTCTCAATGCCAGTGCTTCTTATCTCAGCCTCTCCTGCACTCCTTTACCCCCGTCTCGATGCCAGTGCTTCCTATCTCAGCCTCTCCTGCACTCCT"


motif_embedding_dict = {}
motif_embedding_dict["Random"] = {}
motif_embedding_dict["Random"]["regions"] = utils.random_sequence_by_shuffling(seq_to_shuffle, number_of_random_regions)
```

### (or) Create random sequences that follow the GC-content of the given fasta sequences¶

In [6]:

```
path_to_use_GC_content = "./data/KC_regions.fa"
number_of_random_regions = 100
seq_len = 500


motif_embedding_dict = {}
motif_embedding_dict["Random"] = {}
motif_embedding_dict["Random"]["regions"] = utils.random_sequence_gc_adjusted(seq_len, number_of_random_regions, path_to_use_GC_content)
```

### Define the patterns to be implanted¶

In [7]:

```
patterns_dict = {"Ey": "TGCTCACTCAAGCGTAA",
                 "Mef2": "CTATTTATAG",
                 "Onecut": "ATCGAT",
                 "Sr": "CCACCC"}

for factor in patterns_dict:
    patterns_dict[factor] = utils.one_hot_encode_along_row_axis(patterns_dict[factor])
```

### Here you generate synthetic sequence by in silico evolution.¶

##### **pattern:** selected motif to be implanted¶

##### **region:** initial regions to implant the selected pattern on¶

##### **model:** selected deep learning model¶

##### **class\_no:** chosen class to select the best position to implant motif¶

##### As an output it results a dictionary; **regions** contains the sequences after implantation to the best location and **locations** contains the implanted position as the position of first nucleotide of the implanted motif¶

In [8]:

```
path_to_save = "./output/testdata_motif_implant_100seqs.pkl"


print("Implanting Ey motif to random sequences")
motif_embedding_dict["Ey"] = utils.add_pattern_to_best_location(pattern = patterns_dict["Ey"], 
                                                                regions = motif_embedding_dict["Random"]["regions"], 
                                                                model = model_dict["DeepFlyBrain"], 
                                                                class_no = 35)

print("Implanting Mef2 motif to Ey implanted sequences")
motif_embedding_dict["Ey+Mef2"] = utils.add_pattern_to_best_location(pattern = patterns_dict["Mef2"], 
                                                                     regions = motif_embedding_dict["Ey"]["regions"], 
                                                                     model = model_dict["DeepFlyBrain"], 
                                                                     class_no = 35)

print("Scoring final sequences")
for key in motif_embedding_dict:
    motif_embedding_dict[key]["prediction"] = model_dict["DeepFlyBrain"].predict(motif_embedding_dict[key]["regions"])
    
import pickle
f = open(path_to_save, "wb")
pickle.dump(motif_embedding_dict,f)
f.close()
```

```
Implanting Ey motif to random sequences
Sequence index: 0,1,2,3,4,5,6,7,8,9,10,11,12,13,14,15,16,17,18,19,20,21,22,23,24,25,26,27,28,29,30,31,32,33,34,35,36,37,38,39,40,41,42,43,44,45,46,47,48,49,50,51,52,53,54,55,56,57,58,59,60,61,62,63,64,65,66,67,68,69,70,71,72,73,74,75,76,77,78,79,80,81,82,83,84,85,86,87,88,89,90,91,92,93,94,95,96,97,98,99,
Implanting Mef2 motif to Ey implanted sequences
Sequence index: 0,1,2,3,4,5,6,7,8,9,10,11,12,13,14,15,16,17,18,19,20,21,22,23,24,25,26,27,28,29,30,31,32,33,34,35,36,37,38,39,40,41,42,43,44,45,46,47,48,49,50,51,52,53,54,55,56,57,58,59,60,61,62,63,64,65,66,67,68,69,70,71,72,73,74,75,76,77,78,79,80,81,82,83,84,85,86,87,88,89,90,91,92,93,94,95,96,97,98,99,
Scoring final sequences
```

### Load the saved file¶

In [9]:

```
path_to_file = "./output/testdata_motif_implant_100seqs.pkl"


import pickle
f = open(path_to_file, "rb")
motif_embedding_dict = pickle.load(f)
f.close()
```

### Plot the prediction distributions¶

In [10]:

```
class_no = 35
_ = plt.boxplot([motif_embedding_dict[key]["prediction"][:,class_no-1] for key in motif_embedding_dict],notch=True,showfliers=False,whis=[5,95])
_ = plt.xticks(range(1,len(motif_embedding_dict.keys())+1),list(motif_embedding_dict.keys()))
plt.ylabel("Prediction score")
```

Out[10]:

```
Text(0, 0.5, 'Prediction score')
```

### Plot the location distribution of an implanted motif¶

In [11]:

```
_ = plt.hist(motif_embedding_dict["Ey"]["locations"],bins=500,range=(0,500))
_ = plt.ylabel("Number of sequences")
_ = plt.xlabel("Position (bp)")
_ = plt.title("Location of Ey")
```

### Plot the location distribution of an implanted motif relative to another implanted motif¶

In [12]:

```
_ = plt.hist(motif_embedding_dict["Ey+Mef2"]["locations"]-motif_embedding_dict["Ey"]["locations"],bins=401,range=(-200.5,200.5))
_ = plt.ylabel("Number of sequences")
_ = plt.xlabel("Position (bp)")
_ = plt.title("Location of Mef2 relative to Ey")
```

### Plot deepexplainer and saturation mutagenesis of a selected sequence¶

In [13]:

```
index = 19
class_no =  35
onehot_ = np.copy(motif_embedding_dict["Ey+Mef2"]["regions"][index:index+1])


# Initialize the explainer for the selected class
import shap
tf.disable_eager_execution()
rn=np.random.choice(DeepFlyBrain_data_dict["nonAugmented_data"].shape[0], 250, replace=False)
name = "DeepFlyBrain"
explainer_dict = {} 
explainer_dict[name+"_c"+str(class_no)] = shap.DeepExplainer((model_dict[name].inputs,
                                                              model_dict[name].layers[-1].output[:,class_no-1]),
                                                             DeepFlyBrain_data_dict["nonAugmented_data"][rn])


# Plot contribution scores and saturation mutagenesis
ntrack = 2
fig = plt.figure(figsize=(80,ntrack*5))
ax1 = utils.plot_deepexplainer_givenax(explainer=explainer_dict["DeepFlyBrain_c35"], fig=fig, ntrack=ntrack, track_no=1, seq_onehot=onehot_)
_ = utils.plot_mutagenesis_givenax(model=model_dict["DeepFlyBrain"], fig=fig, ntrack=ntrack, track_no=2, seq_onehot=onehot_, class_no = class_no)
```

### Plot prediction of a region at different steps¶

In [14]:

```
index = 19
class_no = 35

for i,step in enumerate(motif_embedding_dict):
    plt.bar(i,motif_embedding_dict[step]["prediction"][index,class_no-1],label=step,color="gray")
_ = plt.xticks(range(len(motif_embedding_dict.keys())),list(motif_embedding_dict.keys()))
_ = plt.ylabel("Prediction score")
```

### Convert created sequence to letters¶

In [15]:

```
index = 19
onehot_ = np.copy(motif_embedding_dict["Ey+Mef2"]["regions"][index:index+1])

print("Generated sequence:",end=" ")
for nuc in onehot_[0]:
    if nuc[0]==1:
        print("A",end="")
    if nuc[1]==1:
        print("C",end="")
    if nuc[2]==1:
        print("G",end="")
    if nuc[3]==1:
        print("T",end="")
print("")
```

```
Generated sequence: TGACTTATGGGTATCGAGTAAAATAGGCAGAATCTGATGGTGGCTGTATAATATAATATATATCCCAGTATCCCCTGAATTTGTTACTGTTCTAGATTTTAAATCAAAAGCCCCGTCTTTGGCCGGCATATTAAGCTACCAACCTCTTATGGAAGGTTCAGGCCATCCGAGTGGCGATATACCAACTGGGGCTCGGCTGGGTATAATAAGTCGTTTGTCAGTGAGACCGGAACCCGGAGCACTGGACATCTGTACTCGTGTGCGTGGTACCGCTATTTATAGGCCGCATCGTTAATCCGTGCTCACTCAAGCGTAAAATAGACAGAATGGAGAGCTAAGAGGGTAGAACGAGGTTGGCGGTCGTTCTTAAATTATAGACCCATAGTTTGTTAGCCCCTGCGATACACCTCTCTTTCAGGTCGAGTATAGCATCTAGAGCACGTGGGGGTGTCCCTAGTAGTTTAAGTGGGGTACGATTGCCTTTTAATTAGCTTGTCAGT
```

In [ ]:

```

```
